# Thermoanalytical and Tensile Strength Studies of Polypropylene Fibre-Reinforced Cement Composites Designed for Tunnel Applications

**DOI:** 10.3390/ma19010142

**Published:** 2025-12-31

**Authors:** Tomasz Drzymała, Ewa Rudnik, Sylwia Lewicka

**Affiliations:** 1 Faculty of Safety Engineering and Civil Protection, Fire University, 52/54 Słowackiego Street, 01-629 Warsaw, Poland; 2 Institute of Safety Engineering, Fire University, 52/54 Słowackiego Street, 01-629 Warsaw, Poland; erudnik@apoz.edu.pl; 3 Independent Researcher, 41-803 Zabrze, Poland

**Keywords:** cementitious composite, cement mortars, polypropylene fibre, thermal degradation, effect of high temperature, tunnel applications, mechanical properties

## Abstract

In this article, the thermal and mechanical properties of mortars reinforced with polypropylene (PP) fibres have been studied. Particularly, the effect of polypropylene fibres’ addition on the thermal behaviour of fine-grained building mortars at high temperatures was studied using simultaneous thermal analysis. Two types of polypropylene fibres, differing in shape and size, were used as fillers. The thermal behaviour of cement mortar samples with and without fibres was described. Special attention was given to the thermal behaviour of fibre-reinforced cement mortars subjected to the high temperatures of 100 °C, 200 °C, 300 °C, 400 °C, 500 °C, and 600 °C. Comparative studies using simultaneous thermal analysis (STA) were also performed for non-heated samples (20 °C). The TG, DTG, and DTA curves were analysed to investigate the effects related to the dehydration and the decomposition of hydration and carbonation products. Compared to mortar samples without fibres, the results showed that the presence of polypropylene fibres contributes to an increase in the thermal stability of the samples. It has been proven that the impact of the type and amount of PP fibres in the tested range (1.8 kg/m^3^ vs. 3.6 kg/m^3^) on the thermal stability of specimens of tested cement composites was found not to be significantly visible. Next, extensive research was performed on the impact of fire environmental exposure on the variability in the strength parameters of the mortars. Tensile strength tests were conducted based on the standards specified by the Polish Committee for Standardization. The research material consisted of high-strength, fine-grained building mortars, modified by an original method with polypropylene fibres at concentration of 1.8 kg/m^3^, 3.0 kg/m^3^, and 3.6 kg/m^3^. For reference, ordinary mortars without fibres were used, as well. Tensile strength was evaluated for mortar samples, which were exposed to temperatures of 100 °C, 200 °C, 300 °C, 400 °C, 500 °C, and 600 °C, respectively. Special attention was paid to the thermal behaviour of cement mortars reinforced with polypropylene (PP) fibres, subjected to high temperatures. Based on the obtained test results, a detailed statistical analysis was developed, along with comprehensive temperature–parameter relationships, which could enable an approximate post-failure assessment of the mortar’s condition. The main outcomes of this paper include optimal fibre dosage, which is 3.6 kg/m^3^, identified optimal fibre type, namely F fibre, as well as plateau in tensile strength for temperatures between 200 °C and 400 °C for fibre-reinforced samples.

## 1. Introduction

Ensuring safe transport in tunnels, with particular focus on transport tunnels, is one of the most important issues of global significance. In the European Union, special attention has been drawn to this problem due to tragic events characterised by a high number of fatalities and significant social and financial losses. In recent years, numerous initiatives have been undertaken by the European Commission to ensure safety in road and rail tunnels, resulting in the development of new directives and the funding of several national and international projects. It should be noted that the topic of protecting structures from the effects of fire has also been addressed, along with issues related to the potential destruction of tunnel structures and the minimisation of the resulting hazards, particularly those associated with explosive spalling of concrete [1].

Concrete structures offer high fire resistance, which consequently ensures a high level of safety for people, property, and the environment, thus aligning with the new fundamental requirements in the area of sustainable development. However, the impact of fire on structures made of cement composites is the cause of numerous emergency situations. Fires, regardless of whether they occur in public, residential, or industrial buildings or in transport tunnels, give rise to numerous risks, not only during their occurrence and the rescue operations but also during the post-failure operation of these structures. In transport tunnels and other unheated spaces (such as basements and garages), a characteristic feature of concrete is its relatively high moisture content. In the zone of direct fire exposure, the phenomenon of thermal spalling has been frequently observed. This refers to the sudden detachment of fragments from the surface layer of the concrete structure [2,3,4,5,6,7,8,9,10]. This phenomenon is particularly dangerous in the case of structures made of HSC (high-strength concrete), which are characterised by high tightness and low permeability. Concrete spalling reduces the cover of reinforcing bars, exposing the structure to a loss of its load-bearing capacity [11,12]. This poses a serious threat to rescue services and significantly reduces the load-bearing capacity and safety of the structure [13,14,15].

The topic of the processes that occur in cement composites under high-temperature conditions has been thoroughly analysed by a number of scientists [16,17,18,19,20,21,22,23]. Issues addressed in this area of analysis take two seemingly closely related directions. The first concerns cement composites, which are designed to withstand loads at consistently elevated temperatures, as is the case, for example, of the lining of industrial chimneys [24]. The second direction investigates the behaviour of materials for which exposure to high temperatures is only an emergency, as is the case in fires [25].

So far, the most economical and technologically justified method for preventing spalling is the addition of polypropylene fibres. It is also worth mentioning that this method is recommended by Eurocode 2 [2,3]. The use of fibre-reinforced concrete in tunnel linings has increased over the last two decades, particularly in relation to precast segmental tunnel linings and underground structures [26].

A number of scientific and research studies have been dedicated to proving the positive impact of polypropylene (PP) fibres on concrete structures during fire exposure, including those funded by the European Commission, for which safety in European transport tunnels has become the primary concern [1,27]. Based on the results of theoretical and experimental scientific research devoted to explaining the phenomenon of explosive concrete spalling, several theories have been developed [4,7,8,9,10,28,29,30,31]; however, no uniform, consistent explanation of its cause has been obtained. Presently there are two main theories regarding the mechanism of spalling formation. According to the first one, which is primarily accepted by European scientists, the main cause of the explosive spalling of the outer layer of concrete is the high gas pressure generated by the evaporation of moisture in the surface layer of concrete structures, coupled with the simultaneous reduction in the strength of concrete at high temperatures. The second theory, developed principally by American scientists, explains the occurrence of spalling via the high tensile stresses in concrete. The accumulated potential deformation energy is released suddenly once it exceeds the fracture energy of the material. The phenomenon of spalling takes on particular significance during rescue operations in transport tunnels. In tunnel structures, the development of a fire and its consequences are generally far more severe than in other engineering structures [32,33,34]. Rescue operations are highly complex due to numerous difficulties that are related, among other things, to accessibility [35,36].

According to the literature sources [37,38,39,40,41,42], the most likely cause of this phenomenon is the physical processes that occur within the volume of concrete, which are associated with the increasing pressure of water vapour contained in the pore spaces of the concrete. As the temperature rises, the volume of water increases, which exerts pressure on the pore walls, resulting in the formation of tensile stresses within the mentioned zone [43]. When these stresses exceed the tensile strength of the concrete, its fragments break away explosively [2]. This phenomenon applies in particular to high-strength concrete with high impermeability [3], in which water vapour cannot expand freely. There are also hypotheses that the destructive processes and physical changes described above may also occur within the concrete itself and may not be visible from the outside.

Although the effectiveness of fibres can be demonstrated experimentally, the mechanisms preventing explosions are still debatable [44]. From an industrial perspective, to prevent thermal spalling in BWW concretes [9], the following values are accepted: a fibre dosage of 2 kg/m^3^, fibre length of 10–20 mm, and fibre diameter of 50–200 μm. P. Kalifa [9], and Behnood and Ghandehari [45] demonstrate the effectiveness of polypropylene fibres against thermal spalling, even at doses as low as 0.9 kg/m^3^. Many studies present the results of the impact of various fibres on the mechanical and/or physicochemical properties of concrete at normal and elevated temperatures [1,2,3,9,15,46,47,48,49,50,51,52,53,54,55]. Within the subject area of improving the properties of cement composites at normal temperatures and degrading the mechanical properties of cement composites under high-temperature conditions, the recent literature indicates significant variation in their behaviour depending on the type of binder [56,57], microstructure, and the presence of reinforcing fibres [58,59,60,61].

The cited results confirm that applying appropriate fibre types and binder compositions (e.g., with the addition of slag or silica fume) can significantly increase the fire resistance of concrete and mortars, improving their efficiency under extreme temperature conditions. Continuous research into the use of a wide range of admixtures, additives, and diverse cement composite structures is still relevant and justified. Given the above findings, it is particularly important to conduct research on the efficiency of polypropylene fibres in typical construction mortars, which remain the basic material for many structural elements, and also to develop relationships enabling the assessment of their condition after fires [61].

Previous strength tests conducted by [1] were carried out for different fibre contents in the concrete mix (0.6 kg/m^3^; 0.9 kg/m^3^; 1.2 kg/m^3^; 1.5 kg/m^3^). The tests were performed for both monofilament and fibrillated fibres, as well as for different fibre lengths (12 mm and 19 mm) and diameters (18 μm and 40 μm). The tests were carried out on concrete specimens containing coarse aggregate. The research presented in this article was made on cement mortars modified with the addition of polypropylene fibres. This approach allowed for the elimination of the influence of coarse aggregate on the accuracy of the measured strength property. The impact of the high temperature on the development of the physicomechanical and physicochemical properties of the tested cement composites were subjected to an in-depth analysis. The composition of cement mortars is similar to that of concrete. Mortar also consists of cement paste and aggregate, but its particle size is finer. As a result, the temperature distribution within the mortar is more uniform. From the perspective of the potential for damage due to the differential thermal expansion of the various phases or layers of the composite, this aspect should be minimised. The main causes of mortar destruction under thermal load, as identified by the authors, may be their excessive impermeability and the common use of silica sands in the production. The main hypothesis of the proposed research assumed that the modification of high-strength traditional mortars with polypropylene fibres would improve their properties under fire conditions. Moreover, the PP fibres in the tested cement composites positively affect the thermal stability of the cement mortars. Lower mass losses and higher distribution temperatures were observed in the samples containing PP fibres. For this reason, the higher thermal stability of the samples with PP fibres may also play a significant role in the analysis of spalling mechanisms.

Previous findings by the authors [55], using simultaneous thermal analysis, have shown that reinforcing cement mortar with PP fibres leads to an increase in its thermal stability after initial heating at 200 °C and 300 °C. This can be explained by a combination of dehydration of hydration products and melting of PP fibres after heat treatment, which affected the residual permeability of the heated samples and led to changes in the resulting structure.

The aim of the work was to apply the combined simultaneous thermal analysis (STA) and tensile tests in order to elucidate the role of polypropylene fibres in high-strength, fine-grained mortars modified with polypropylene fibres exposed to fire environments. Special emphasis was given to the detailed statistical analysis of the thermoanalytical and mechanical results. According to authors’ best knowledge, such an approach has not been described in the literature.

Various types of polypropylene fibres were used in the tests. The tested polypropylene fibres differed in form (monofilament versus fibrillated, in a bundle) and size. The tests were carried out on mortars reinforced with polypropylene fibres in quantities of 1.8 kg/m^3^, 3.0 kg/m^3^, and 3.6 kg/m^3^ and unmodified (control samples).

## 2. Materials and Methods

### 2.1. Materials

CEM I 42.5 R Lafarge cement was used to make the mortar, which, according to the manufacturer’s declaration, meets the requirements of the standard PN-EN 197-1:2012, “Cement. Part 1: Composition, requirements and conformity criteria for common cements” [62]. This cement is characterised by stable physicochemical properties, appropriate setting time, high early and final strength parameters, low alkali content, and high resistance to aggressive chemical agents, which is why it is commonly used in the production of commercial concrete mixtures. The detailed values of the physicochemical parameters of the cement are presented in Table 1.

Vistula sand (0/2 mm) and Silimic silica fume were also used for the mortar. For practical reasons, washed sand with a fraction of 0–2 mm was used for mortars. This type of sand is characterised by a high compaction coefficient and granularity, and is mostly used in construction for preparing concrete and mortar. Silica fume is a by-product obtained during the manufacture of metallic silicon and ferrosilicon alloys in electric arc furnaces. It consists of fine particles, with a diameter estimated to be on average one hundred times smaller than the average particle size of cement. Replacing 15% of the cement with silica fume, according to the manufacturer’s data, increases the impermeability of the concrete by several dozen times, which is difficult to achieve via other methods. Additionally, the compressive strength increases by 20%, and the water absorption is reduced threefold. In the context of modifying composites with silica fume that may be exposed to high temperatures, this addition is considered unfavourable. The increase in the impermeability of the cement composite hinders the free evaporation of water contained in the capillaries. The water, which increases in volume, can generate tensile stresses in the walls of the capillaries and, once the tensile strength is exceeded, lead to the destruction of the composite. In order to take advantage of the original properties of microsilica, it was decided to use it. As an additive to concrete, microsilica has a number of beneficial properties that significantly improve its parameters and durability. It primarily improves strength, seals the structure, increases resistance to external factors and corrosion, and also enhances the self-compacting properties of the concrete mix. The basic properties of silica fume, as taken from its technical datasheet, are presented in Table 2.

Tap water that was compliant with the requirements of [66] PN-EN 1008:2004, “Mixing water for concrete. Specification for sampling, testing and evaluation of mixing water for concrete” was used for the mortar. Chrysofluid Optima 185 (Chryso Saint Gobain, Courbevoie, France) was used as an admixture in accordance with the requirements of [67] PN-EN 934-2, “Admixtures for concrete, mortar and grout. Part 2: Admixtures for concrete. Definitions, requirements, conformity, marking and labelling”. Chrysofluid is a high-performance plasticiser. It is mainly used in the production of high-strength commercial concrete, and self-compacting and easy-to-compact mixes. According to the manufacturer’s claims, it plasticises and homogenises the concrete mix, maintains consistency over a long period, and enhances the early strength of the concrete. The basic properties of the admixture, as taken from its technical datasheet, are presented in Table 3.

For the tests, two types of polypropylene fibres, also used as concrete additives, were employed, designated for the purposes of this study, as I and F, respectively. Polypropylene fibre I, with the trade name Ignis^®^ (Schomburg Rethmeier, Detmold, Germany), and fibre F, with the trade name Fortatech^®^ Fibre High Grade 190 (currently Fibrofor^®^ Fibre High Grade 190, Contec Fiber, Domat/Ems, Switzerland), were used. The fibres differed primarily in the length and thickness of individual filaments. Fibre I is a monofilament fibre, meaning it is composed of a single strand. Fibre F is a multifilament fibre, i.e., it is formed by twisting several individual strands together. Polypropylene fibres I and F serve as dispersed micro-reinforcements in the form of filaments with lengths of 12–19 mm and diameters of 18–40 μm, respectively. Their addition was intended to strengthen the structure of the unheated composites, and they functioned as dispersed reinforcement. In composites exposed to high temperatures, the fibres were expected to preserve the integrity of the mortar. The channels formed after their melting were intended to facilitate the evaporation of residual capillary water.

**Figure 1:**
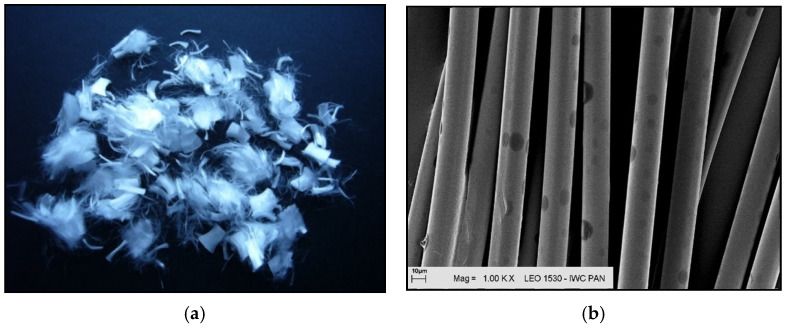
Polypropylene fibres ‘I’: (**a**) photo without magnification; (**b**) SEM photo.

**Figure 2:**
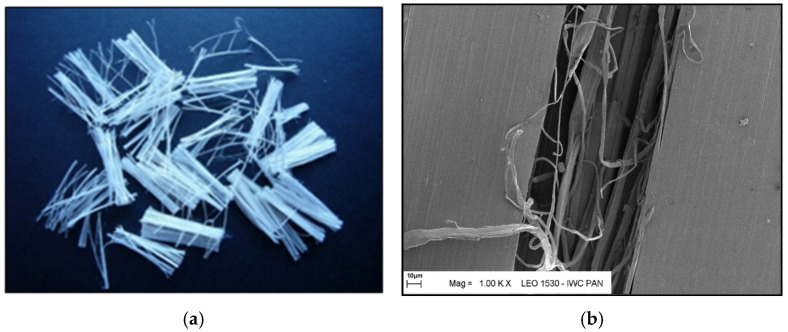
Polypropylene fibres ‘F’: (**a**) photo without magnification; (**b**) SEM photo.

Additional information on the shape and structure of polypropylene fibres was obtained through observations using a scanning electron microscope (SEM). Polypropylene fibres “I” examined under SEM exhibited relatively smooth surfaces; only at higher magnifications, on the order of 5000×, were a few protrusions or bubbles, ranging in size from 2 μm to 10 μm, visible, randomly distributed on the fibre surfaces. The diameters of the fibres, measured randomly, ranged from 17.8 μm to 19.6 μm. The polypropylene fibres “F”, examined under SEM, are characterised by a layered structure and tend to separate relatively easily into very fine filaments. For this reason, it is difficult to distinguish individual fibres; rather, they can be treated as fibre bundles. Consequently, it is also challenging to accurately measure the diameter of these fibres or bundles. Two types of polypropylene fibres were used: monofilament Ignis (abbreviation: I), and fibrillated (abbreviation: F). The images of the PP fibres are given in Figure 1 and Figure 2.

Polypropylene fibres “I” with the trade name Ignis^®^ (Shomburg Rethmeier, Detmold, Germany), according to the manufacturer’s description, enable crack reduction and reduce shrinkage, thereby improving the surface properties and durability of concrete elements. Fibres “I” are used across a wide range of applications, including the construction of railway and road tunnels, high-risk structures, cable tunnels, bridges, underground car parks, and refractory products. The addition of polypropylene fibres “I” to a concrete mix provides passive fire protection, enhancing the durability of concrete elements during a fire.

Polypropylene fibres “F” with the trade name Fortatech^®^ Fibre High Grade 190 (currently Fibrofor^®^ Fibre High Grade 190, Contec Fiber, Domat/Ems, Switzerland), according to the manufacturer’s description, are fibrillated, high-quality, bundled fibres used as structural reinforcement in concrete. Due to their rough surface, they bond effectively with the concrete and are rapidly and three-dimensionally dispersed within the matrix during mixing of the concrete components. Fibres “F” prevent plastic shrinkage and enhance the impact resistance of concrete, as well. In addition, they reduce the settlement of concrete mix components and increase the durability of concrete in structures exposed to aggressive waters. Table 4 specifies the properties of the polypropylene fibres according to the manufacturers’ information.

### 2.2. Sample Preparation

The specimens for testing were prepared at the Concrete Technology Laboratory of the Division of Building Materials Engineering, Faculty of Civil Engineering, the Warsaw University of Technology. Prior to the commencement of testing, cement mortar compositions were designed with and without the addition of polypropylene fibres. Within each test series, mortar specimens were prepared based on the same mix design, which is presented in Table 5.

The model research material was cement mortar with a low water-to-binder ratio, sealed with the addition of the described silica fume. Its composition was provided by the contracting company that uses it for the production of building products. The used admixture enabled the production of a cohesive concrete mix with low viscosity and a very long-lasting effect of maintaining consistency. The research presented in the article focused on an analysis of thermal changes and the assessment of the possibility of improving the strength parameters of the tested cement composites, subjected to high temperatures, by introducing polypropylene PP fibres into the mixture. The composition of the base mortar was therefore modified by adding polypropylene fibres in the following quantities: for thermal change tests, 1.8 kg/m^3^ and 3.6 kg/m^3^; and for strength tests, 1.8 kg/m^3^, 3.0 kg/m^3^ and 3.6 kg/m^3^.

All of the mortar mixtures were prepared in a laboratory mixer. First, the dry ingredients (sand and cement) were added and mixed for about 60 s. Then, the polypropylene fibres were added and mixed for 30 s (this short mixing time was due to the fact that longer mixing led to the fibres clumping together and settling on the mixer blades). After that, water and the admixture (2% of the cement mass) were added. All the ingredients were mixed for an additional 3 min. The specimens were formed and compacted in two layers on a vibrating table for approximately 15 s. The moulded specimens were then placed on a level laboratory floor and tightly covered with foil to prevent excessive evaporation of water from their surfaces. After 24 h, the specimens were demoulded and immersed in water for the next 27 days. Subsequently, the specimens were stored in a climatic chamber for 60 days at a temperature of 20 °C and a relative humidity of 99%. The specimens were then placed in a drying oven and dried to a constant mass at a temperature of 105 °C, while monitoring water loss throughout the process. The presented method of drying (where the mortar samples dry to a constant mass before the heating process) was manufactured in order to prevent “spalling” explosions which would result in damage of the experimental set-up. Such phenomena were recorded during preliminary studies, especially when heating the mortar samples to a temperature of 600 °C. Subsequently, each specimen was thoroughly documented, including the date of its casting, and assigned a unique identification number (each number corresponded to a specific test recorded on the control sheet, thus preventing potential errors). All the specimens were formed and stored in an identical manner until testing. The testing methodology was consistent with the procedure used in [55].

### 2.3. Procedure of Thermal Treatment of Samples

**Figure 3:**
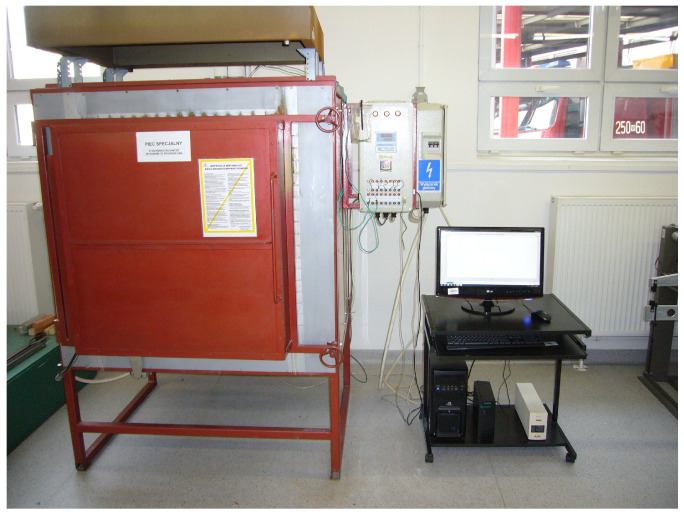
Sample heating station.

The fire environment simulation was conducted at the Applied Mechanics Laboratory at the Fire Academy. The sample heating station consisted of an electric medium-temperature chamber furnace of the type PK 1100/5, along with a PC (Figure 3). The furnace was controlled and the temperature during the heating of the samples was recorded using specialised software. The furnace frame was made of square pipes and stainless steel sheets. The furnace insulation layer consisted of ceramic fibre blocks and mats. The heating elements of the furnace chamber was made of KANTHAL A1 resistance wire in the form of spirals. Additionally, the furnace ceiling was equipped with two flues for steam discharge or the introduction of additional thermoelectric sensors. The control thermocouple (TR) was introduced through the back wall of the furnace and positioned near its ceiling. The furnace control system was housed in two cabinets connected to the furnace’s load-bearing frame. These cabinets managed the temperature regulation and the transmission of thermoelectric signals from the measurement sensors to the computer.

**Figure 4:**
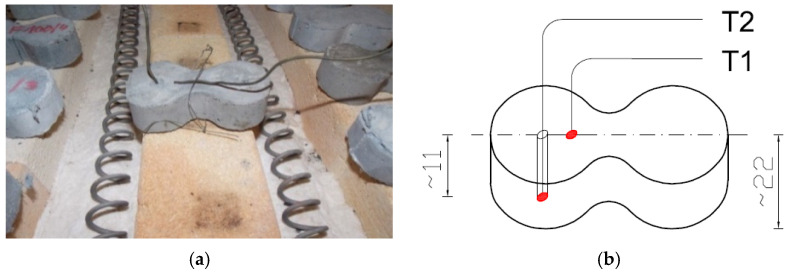
Arrangement of measuring thermocouples in an octagonal sample: (**a**) view of the sample with thermocouples attached; (**b**) diagram of the arrangement of thermocouples in the sample [55].

After the designated conditioning period, the specimens of each composite were divided into groups and subjected to the heating process. The test temperatures ranged from 20 °C to 600 °C. The specimens were heated in a furnace at six target temperatures: 100 °C, 200 °C, 300 °C, 400 °C, 500 °C, and 600 °C. During the tests, efforts were made to ensure that the temperature profile over time approximated the thermal conditions of a standard fire. The heating process and temperature monitoring (allowing registration at a frequency of up to 1 Hz) were carried out using dedicated software from Thermolab S.C. (Warsaw, Poland). The ThermoPro v. 1.2 SGSP programme enables the programming of arbitrary temperature profiles over the duration of the test, the maintenance of specified temperatures for set periods, and the control of the heating rate to reach the desired temperature. Type K thermocouples (NiCr–NiAl), compliant with the standard [68] PN-EN 60584-1:2014-04, were used for temperature measurement. The standard precisely defines the relationship between temperature and the thermoelectric voltage generated for each type of thermocouple. For the measurement of temperature during the heating of the specimens, three thermoelements were used: a control thermoelement (TR) measuring the temperature inside the furnace, and two thermoelements measuring the temperature within the specimen (T1, T2). Thermoelement T1 was attached to the specimen’s wall, while the second thermoelement (T2) was placed in a drilled channel (Figure 4). The tip of this thermocouple was positioned at half the specimen height. The depth of thermoelement placement was 11 mm for the “eight-shaped” specimens. In each case, in addition to the specimen equipped with the measurement thermoelements, a batch of 13 specimens was heated (three specimens without fibre addition and three specimens with fibre addition at 1.8 kg/m^3^, 3.0 kg/m^3^, and 3.6 kg/m^3^, respectively). The temperature of the additional specimen was measured using the thermoelements T1 and T2. This prepared batch of specimens was then subjected to heating at six target temperatures (100 °C, 200 °C, 300 °C, 400 °C, 500 °C, and 600 °C). The temperature of 20 °C was assumed as reference.

**Figure 5:**
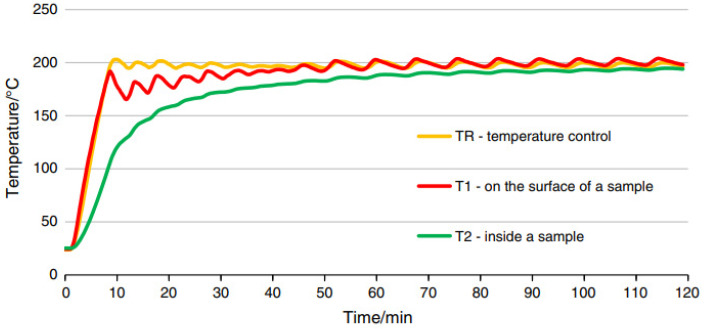
Example of heat treatment of mortar sample at 200 °C [55].

The heating of the specimens was performed according to the developed thermal treatment methodology. The specimens were heated following a curve representing the temperature rise during a standard fire within a concrete element. The procedure was carried out in accordance with the guidelines of PN-EN 1991-1-2 [69], “Eurocode 1: Actions on structures; Part 1-2: Actions on structures during fire” (Polish Committee for Standardization, Warsaw, 1991). The samples were heated until the thermocouples reached the target temperature in approximately 120 min. For each target temperature, the heating process was carried out until the temperatures measured by the thermoelements (TR, T1, and T2) stabilised. Subsequently, the set temperature was maintained for an additional 30 min. The heating methodology was identical for all tests. Figure 5 shows an example of the curve illustrating the actual temperature distribution in an “eight-shaped” specimen at the locations of the measurement thermoelements.

After each heating cycle, the furnace was switched off and allowed to cool to a safe temperature (approximately 100 °C). The furnace was then opened, and the specimens were allowed to cool for approximately 24 h until reaching room temperature, defined as 20 °C. Once cooled, the specimens were subjected to strength tests in accordance with the established testing procedure.

### 2.4. Thermal Analysis

Polypropylene fibres were characterised using a differential scanning calorimeter (DSC), Q200 (TA Instruments, New Castle, DE, USA). The following procedure was applied: a first cycle, which involved heating at 10 °C/min from 20 °C to 350 °C; a second cycle, which involved cooling from 350 °C to 20 °C at 10 °C/min; and a third cycle, which involved heating at 10 °C/min from 20 °C to 350 °C.

The thermal analysis of polypropylene fibres and PP fibre-reinforced mortar samples was carried out using a simultaneous TGA/DSC unit, model SDT Q600, from TA Instruments (New Castle, DE, USA). The samples were heated from 30 °C to 1000 °C at a constant rate of 10 °C/min in the air atmosphere (air flow: 100 L/min). Experimental parameters such as heating rate, mass sample, and atmosphere may significantly affect results—for example, mass sample can influence signal-to-noise, sensitivity, and heat flow. The parameters were chosen according to best practices of ISO standards: ISO 11357 Series (Plastics—Differential Scanning Calorimetry) [70], and ISO 11358 (Plastics—Thermogravimetric Analysis) [71]. For the STA measurements, 30 samples were taken from tensile strength mortar samples. Small pieces were crumbled out of the tensile strength mortar sample and put into sample pan of STA instrument. The sample mass was ca. 30 mg.

### 2.5. Mechanical Testing

**Figure 6:**
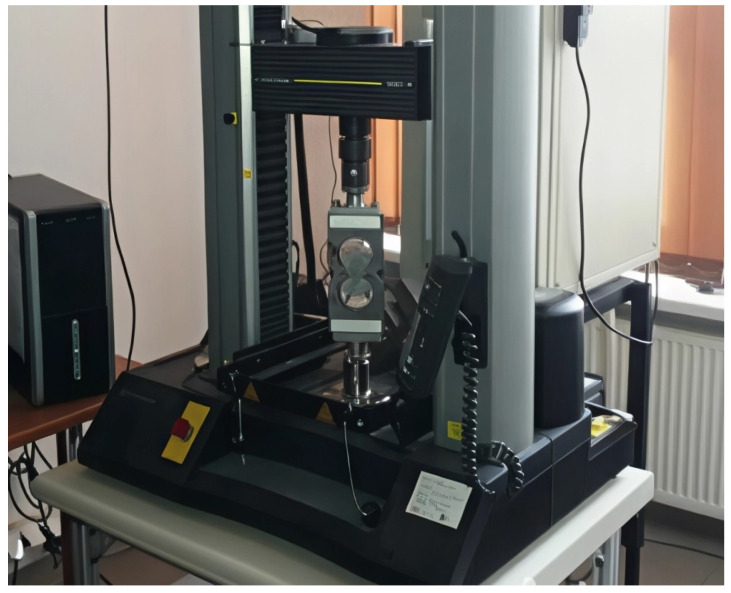
Tensile strength testing station.

The tensile strength test (*f_tm_*) was carried out using an INSTRON 5567 testing machine (INSTRON, Norwood, MA, USA) in accordance with the procedure described in the standard (PN-85/B-04500), “Construction mortars—Testing of physical and mechanical properties” [72]. The testing machine, with a measurement range of 0–30 kN, was equipped with arch-shaped grips for securing the “eight-shaped” specimens (Figure 6). The “eight-shaped” specimens for testing were prepared in special demountable steel moulds that complied with the standard requirements.

## 3. Results and Discussion

### 3.1. Thermal Analysis of Polypropylene Fibres

At the beginning of the study, polypropylene fibres were characterised by thermal analysis methods.

The thermal transitions of polypropylene fibres were analysed by the DSC method.

The endothermic effect corresponding to melting of polypropylene was observed at 161.8 °C and 164 °C, respectively, for Ignis fibres and Fibrofor fibres. Additionally, for Fibrofor fibres, the endothermic peak was observed at 129 °C (Table 6). The initial crystallinity of both fibres was similar, at 27.4% and 25.7%, respectively, for the Ignis and Fibrofor fibres.

**Figure 7:**
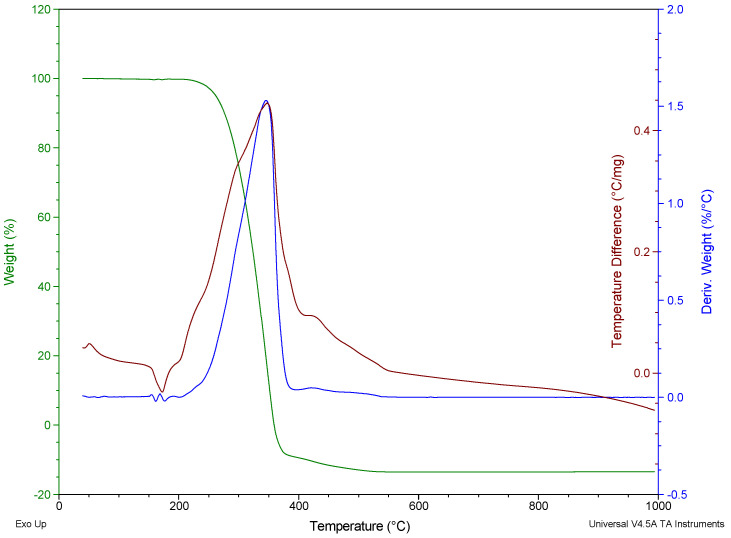
TG/DTG/DTA curves of the Ignis polypropylene fibres in air atmosphere.

**Figure 8:**
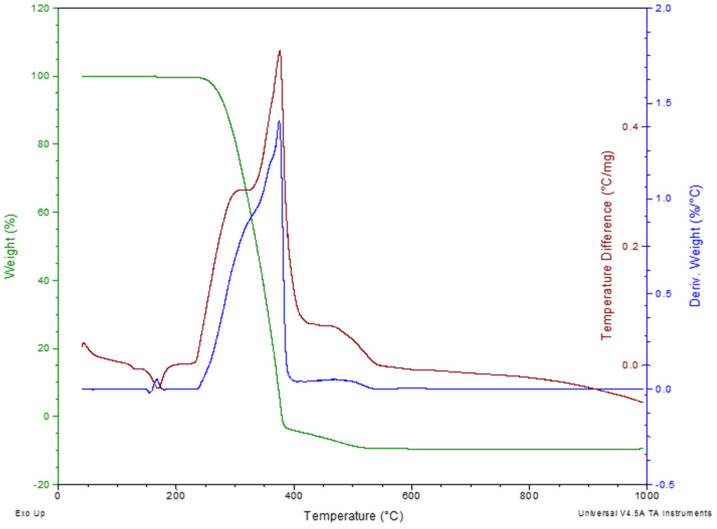
TG/DTG/DTA curves of the Fibrofor polypropylene fibres in air atmosphere.

The thermal degradation of polypropylene fibres was studied in air atmosphere using the simultaneous thermal analysis (STA) method. TG, DTG, and DTA curves of Ignis fibres were given in Figure 7 and Figure 8, respectively, for the Ignis fibres and Fibrofor fibres.

The thermal degradation pattern was similar for both fibres; the degradation proceeded in a single stage within the range 250–400 °C.

The results of thermogravimetric studies of polypropylene fibres using STA method have been summarised in Table 7.

The onset degradation temperature, based on the determination of the temperature at which 5% mass loss occurred, is 261.7 °C for Ignis fibres, and for Fibrofor fibres, it is 10 °C higher.

The main degradation stage of polypropylene fibres proceeds at a temperature above 300 °C. The DTG peak for the Ignis fibres occurs at 345.2 °C, while for the Fibrofor fibres the DTG peak takes place at 374.9 °C.

### 3.2. Thermal Analysis of Polypropylene Fibre-Reinforced Mortar

**Figure 9:**
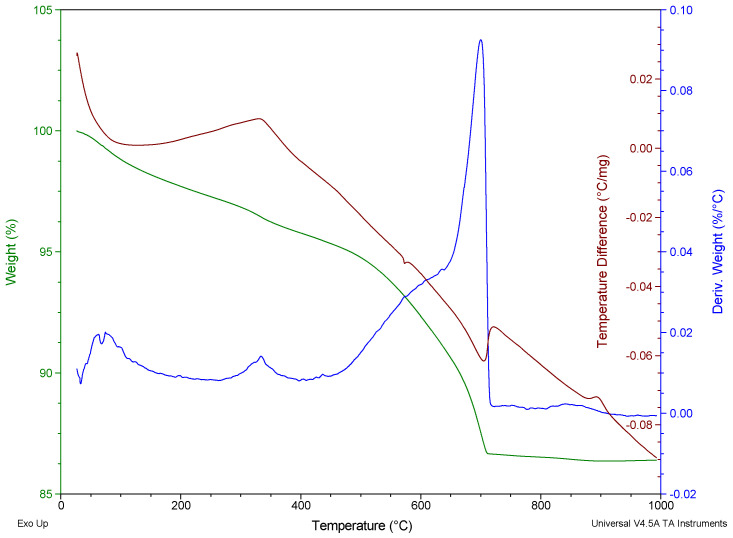
TG/DTG/DTA curves of 1.8 wt % Fibrofor polypropylene fibre-reinforced mortar after thermal treatment at 200 °C.

The thermal properties of polypropylene fibre-reinforced mortar samples were studied by simultaneous thermal analysis (STA) in an air atmosphere. This study concerns samples after thermal treatments at 100 °C, 200 °C, 300 °C, 400 °C, 500 °C, 600 *°C,* and 700 °C. For comparison, polypropylene fibre-reinforced mortar samples not subjected to thermal treatment, as well as mortar sample without fibres, were also studied. The following exemplifies the abbreviations used for samples: in 1.8F/200, 1.8F is the amount (1.8 kg/m^3^) and type of PP fibres (Fibrofor), whereas 200 is the temperature of the heat treatment (200 °C). Examples of the TG/DTG/DTA curves of polypropylene fibre-reinforced mortar are given in Figure 9.

The degradation process can be divided into three stages based on the DTG profile.

The first step between 35 °C and 200 °C is related to the drying of water and/or with the dehydration of ettingrite. At higher temperatures, the DTG peak in the range 340–470 °C corresponds to the dehydroxylation of the calcium hydroxide Ca(OH)_2_. The third mass loss step at ca. 700 °C can be attributed to the decomposition of calcium carbonate, CaCO_3,_ and other carbonates present in the initial cement composition due to the loss of CO_2_ [55].

In Table 8, the mass losses corresponding to thermal effects in mortar have been summarised. The mass loss related to the evaporation of water is 0.2–2.7%. The endothermic effect corresponding to CSH dehydration between 100 and 450 °C accompanies the mass loss of 0.9–3.99%. The mass loss in the temperature range 450–520 °C corresponding to Ca(OH)_2_ decomposition is at a similar level. Moreover, the mass loss corresponding to carbonate decomposition, i.e., 600–900 °C, is in the range of 2.9–10%. The residue at 1000 °C reaches 84–93%. It is noteworthy that the mass loss in the range 600–900 °C for the mortar samples containing polypropylene fibres after the heat treatment is smaller than for the mortar samples without PP fibres.

This pattern can be observed regardless of the kind and amount of polypropylene fibres present after heat treatment 100, 200, 300, 400, and 500 °C, except 600 °C. It seems that there is relationship between carbonate decomposition and thermal stability of polypropylene fibre-modified mortar. However, it requires further investigation.

Table 9 contains a summary of the mass losses up to a given temperature for polypropylene-fibre reinforced mortar samples after heat treatments at 100, 200 °C, 300 °C, 400 °C, 500 °C, and 600 °C.

**Figure 10:**
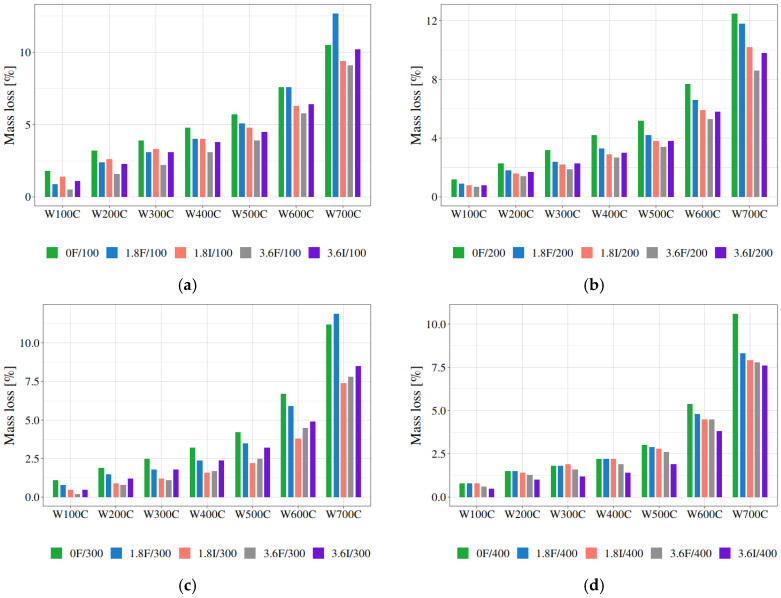
Mass losses of PP-reinforced mortar samples and mortar sample without fibres after heat treatments at (**a**) 100 °C, (**b**) 200 °C, (**c**) 300 °C, and (**d**) 400 °C.

Polypropylene fibre-reinforced mortar samples subjected to heat treatments at 100, 200, 300, and 400 °C exhibit smaller mass losses in comparison to mortar samples without fibres after heat treatments (cf. Figure 10). Moreover, this behaviour is similar to all polypropylene-reinforced mortar samples, regardless of kind and amount of PP fibres. It can be observed that PP fibre-reinforced mortar samples after heat treatment at 500 and 600 °C did not follow this pattern.

### 3.3. Mechanical Properties of Polypropylene Fibre-Reinforced Cement Composite (Cement Mortar)

The laboratory tests were based on the developed experimental plan. The results of the tensile strength determination are presented in Table 10. The table also includes the calculated mean value and the standard deviation of the specimens, which serves as a measure of the uncertainty of the determined mean value.

It can be seen from Table 10 that within the uncertainty of the calculated mean value that the following occurs:

- The strength of the mortar decreases with increasing temperature, with the tensile strength at 600 °C being five-fold lower than at room temperature for all types of mortar: without polypropylene fibres, and with the addition of type I or F fibres at each of the applied concentrations;
- There is a noticeable difference between the strength of fibre-reinforced mortar (particularly at dosages of 3.0 kg/m^3^ and 3.6 kg/m^3^) and that of mortar without fibres;
- Practically, there is rather little difference in the tensile strength of the specimens depending on the fibre concentrations; at most temperatures, there does not seem to be a convincing difference among concentrations of 1.8 kg/m^3^, 3.0 kg/m^3^, and 3.6 kg/m^3^. However, statistical tests provide some information for mortar samples with added F fibre at 3.6 kg/m^3^, but not at a high confidence level (which proves that more studies should be performed). This is particularly evident at higher temperatures, which aligns with the general properties of polypropylene fibres: they soften at temperatures below 200 °C and melt above 300 °C;
- To some extent, the addition of fibres improves the mechanical properties of the mortar; however, above 500 °C all the specimens behave similarly, exhibiting a significant reduction in tensile strength (even more than five-fold). Nevertheless, within the margin of uncertainty, the strength of the specimens with added fibres is higher, although only by 5–7% for type I fibres and 5–10% for type F fibres at dosages of 3.6 kg/m^3^ and 3.0 kg/m^3^, or even up to 19% for type F fibres at a dosage of 1.8 kg/m^3^.

The above preliminary analysis translates into specific conclusions based on statistical analysis. This analysis is somewhat limited due to the small amount of data when the results are grouped according to temperature values (20, 100, 200, 300, 400, 500, and 600 °C) and the density of the particular type of fibre (Z0, Z1.8x, Z3.0x and Z3.6x, where x = I or F). This makes it hard to obtain solid, statistically significant conclusions.

### 3.4. Statistical Analysis of Thermoanalytical and Mechanical Results

**Figure 11:**
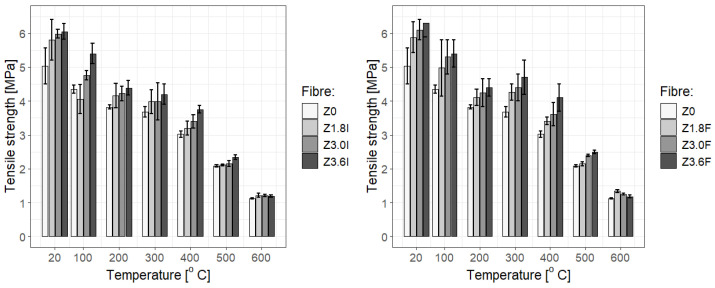
Mean value with std errors for tensile strength of mortar samples with no added PP fibres or with added fibres with fixed density: Z0—no fibre added, Z1.8x (sample with added fibre in 1.8 kg/m^3^ density), Z3.0x (sample with added fibre in 3.0 kg/m^3^ density), and Z3.6x (sample with added fibre in 3.6 kg/m^3^ density); x—F or I.

**Figure 12:**
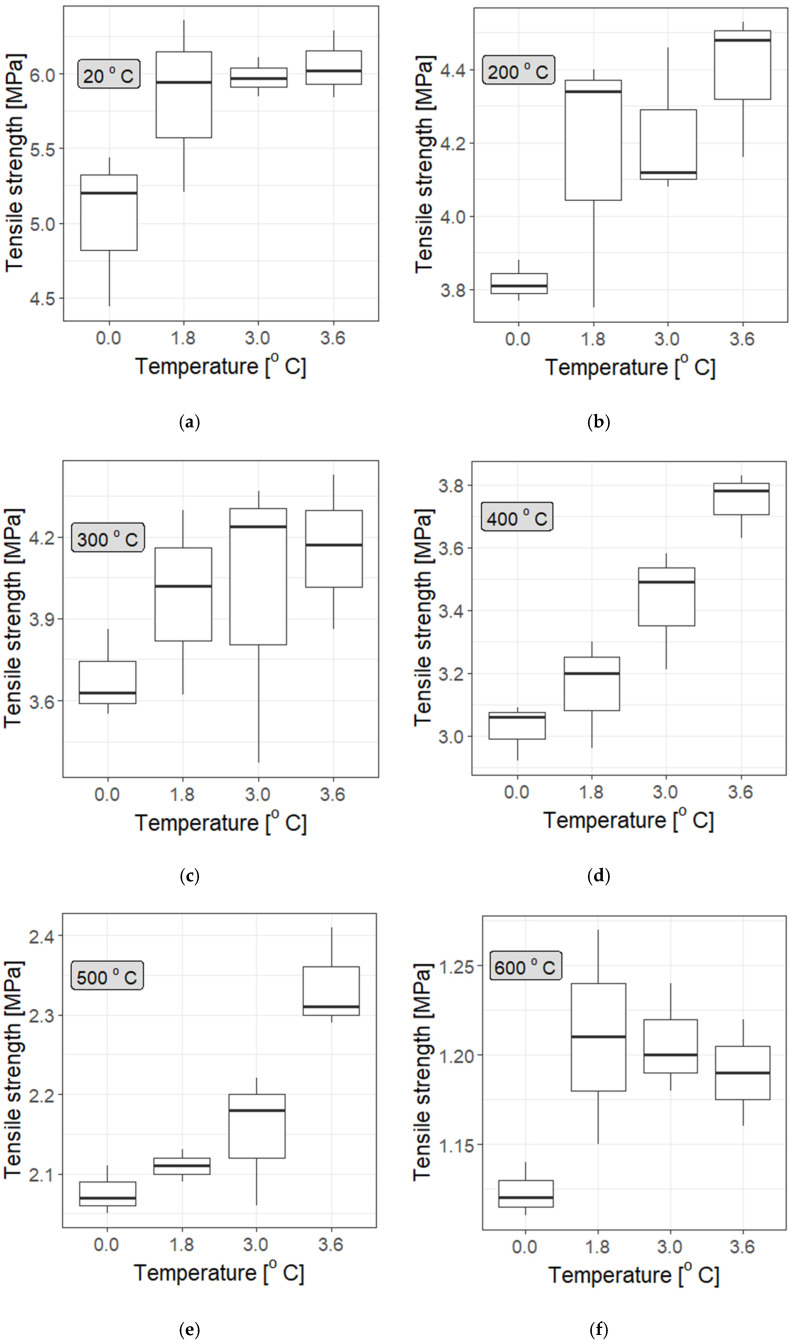
Statistical distributions in the form of box-and-whisker plots for the measured tensile strength of cement mortars with added Ignis fibres at different temperatures: (**a**) 20 °C, (**b**) 200 °C, (**c**) 300 °C, (**d**) 400 °C, (**e**) 500 °C, and (**f**) 600 °C. A significant improvement in strength at room temperature is evident, which is insensitive to fibre dosage (saturation effect at 20 °C, 200 °C, and 300 °C), while at higher temperatures, a dependence on dosage is observed. It should be noted that the initially significantly higher strength of mortars with different fibre contents is maintained throughout the temperature range especially for the 3.6 kg/m^3^ dosage.

**Figure 13:**
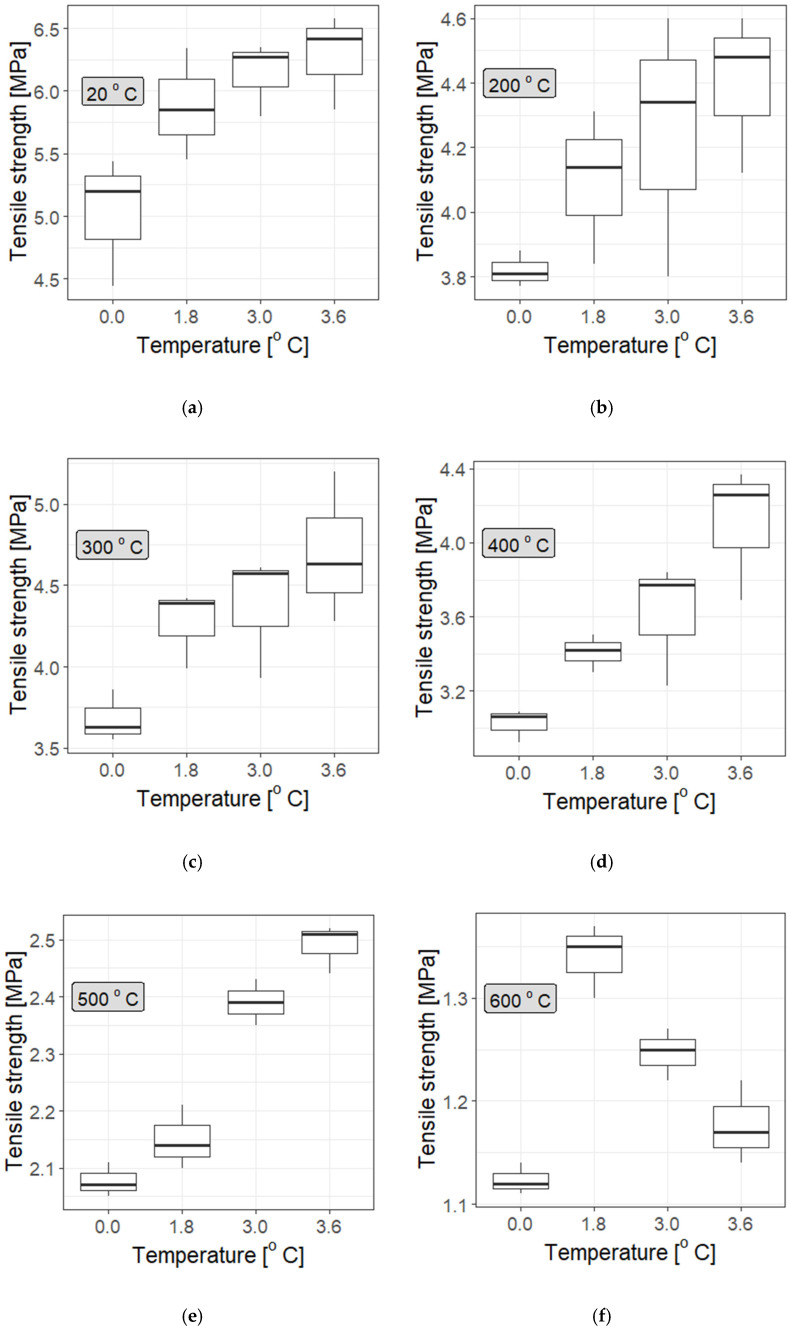
The statistical distribution in the form of box-and-whisker plots for the measured tensile strength of cement mortars with added Fortatech fibres at various temperatures: (**a**) 20 °C, (**b**) 200 °C, (**c**) 300 °C, (**d**) 400 °C, (**e**) 500 °C, and (**f**) 600 °C. It is notable that, except for the 600 °C temperature, in every tested temperature, there is a clear increase in strength as the fibre density increases, for all studied concentrations.

It is evident that the addition of polypropylene fibres improves the strength of cement mortars over a fairly wide temperature range. However, it would be exaggerated to conclude that more fibres necessarily lead to better results (though at some temperatures the improvement in mechanical properties is visible: see, e.g., Z3.0F at 400 °C in Figure 11). Figure 11, Figure 12 and Figure 13 indicate only a slight increase with fibre concentration, and at different temperatures (particularly high temperatures) they show a tendency towards saturation; i.e., increasing the fibre dosage does not significantly improve the outcomes. This outcome is particularly clear in Figure 12 and Figure 13, as well as in the statistical tests summarised in Table 11, although the saturation effect is not uniform at every tested temperature, and it is generally more pronounced for type “I” fibres.

To statistically analyse the results of tensile strength measurements of mortars with and without added fibres, statistical analysis was performed (for the documentation of the software utilised in the study, see [73], and for some basics and a good introduction, please refer to [74]) and, in particular, statistical tests were conducted. The principle of statistical testing is that, based on the obtained results, a so-called null hypothesis is verified at a pre-defined significance level, most commonly (1 − α)·100% = 95%. If the null hypothesis cannot be rejected based on the collected data and the performed test, this does not imply that it can be accepted. At this stage, it can only be assumed that no evidence was found to reject the null hypothesis. However, if the results and the test indicate that the null hypothesis can be rejected, this is always in favour of the alternative hypothesis; hence, statistical tests are often constructed to reject the null hypothesis in favour of the alternative [74,75,76]. Therefore, also in this case, the null hypothesis was adopted, indicating that the tensile strength of mortars with fibres at different dosages is lower than the strength of samples without fibres. Table 11 summarises the results of the *t*-test comparing the mean values for the pairs: cement mortar with added F fibres vs. without fibres, and with I fibres vs. without fibres. The significance level was set at 95% (i.e., α = 0.05), and the test results should be interpreted such that if the calculated *p*-value is less than α, the null hypothesis should be rejected in favour of the alternative hypothesis. This situation occurs over the largest temperature range for the 1.8 kg/m^3^ dosage for both types of fibres, and for 3.0 kg/m^3^ of F fibres and partially for I fibres (see results highlighted in bold or italics in Table 11). Based on the results, it can be concluded that, statistically, the best tensile strength compared to cement mortars without fibre addition is achieved with the addition of fibre F at a minimum of 3.0 kg/m^3^ (and ideally 3.6 kg/m^3^), as well as (with a slightly weaker overall result) the addition of fibre I at a density of 3.6 kg/m^3^.

A similar conclusion can be drawn by performing a statistical test as in the above analysis, but without conditioning on temperature. In that case, the *p*-value will be smaller than the assumed alpha = 0.05 for all fibre F densities and for the densities of 3.0 kg/m^3^ and 3.6 kg/m^3^ of fibre I, see Table 12. The results highlighted in bold in Table 12 suggest that significantly higher strength is achieved for samples with the addition of fibre F at a density of at least 1.8 kg/m^3^, and for fibre I from a density of 3.0 kg/m^3^ upwards. Furthermore, there are statistically significant differences between the densities 1.8 kg/m^3^ and 3.6 kg/m^3^ for both types of fibres, and this leads to a conclusion that a two-fold increase in the content of fibre F or fibre I statistically increases the strength of the mortar.

However, it should be noted that *t*-tests are parametric tests, which are used when the assumption of normality is met, i.e., when measurements follow a Gaussian distribution. To statistically verify this, the Shapiro–Wilk test is performed, also at a significance level of α = 0.05, where the null hypothesis is that the distribution is normal [77,78]. All the data sets—tensile strength for cement mortar without fibre addition and with the addition of fibre F or fibre I for each density—were found to pass the Shapiro–Wilk test, i.e., the null hypothesis of normal distribution cannot be rejected. The corresponding *p*-values are as follows: Z0: *p* = 0.215, Z1.8F: *p* = 0.1, Z1.8I: *p* = 0.214, Z3.0F: *p* = 0.318, Z3.0I: *p* = 0.23, Z3.6F: *p* = 0.244, and Z3.6I: *p* = 0.378.

**Figure 14:**
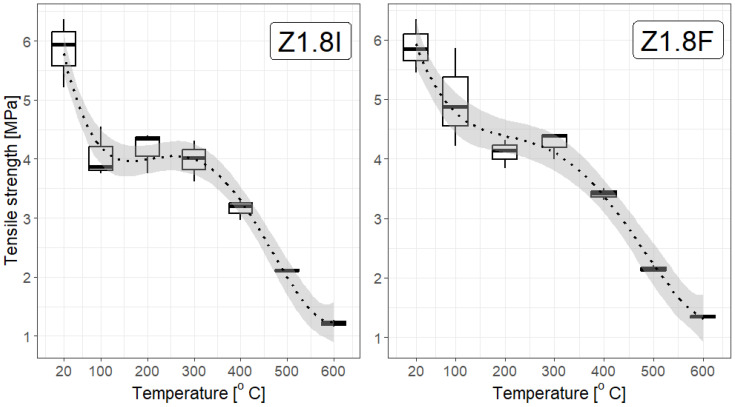
Box plots for the tensile strength of a selected type of fibre with a density of 1.8 kg/m^3^. The linear model of decline with temperature poorly reflects the actual relationship (R^2^ approximately 0.8); therefore, a third-degree polynomial model was used, as justified in the text. The coefficient of determination in this case is 0.94, and the 95% confidence interval is narrow.

Since, based on Figure 14, it appears that the addition of fibre “F” results in mortars being slightly more durable than those with fibre “I”, especially at higher temperatures, statistical tests were conducted to compare these two data sets as well. To do this, a one-sided *t*-test was used for the difference in tensile strength for a given density of fibre F and I, and the null hypothesis that this difference is negative was verified. Where the *p*-value was smaller than the assumed significance level α, the null hypothesis could be rejected in favour of the alternative hypothesis, that the tensile strength for the addition of fibre F is significantly higher than for fibre I. The results of the *t*-test show that significantly better tensile strength is achieved at higher temperatures upon the addition of fibre F; see Table 13. However, before performing the test for the difference in tensile strength for fibre F and I, it was necessary to first check if this could be performed. This can be performed if the variances of both quantities are not significantly different—for this purpose, an F-test was conducted, see [77,79]. At the significance level of α = 0.05, the hypothesis of the homogeneity of variances was tested. The *p*-values indicate that this hypothesis could not be rejected. The obtained values are presented in the third column of Table 13.

**Figure 15:**
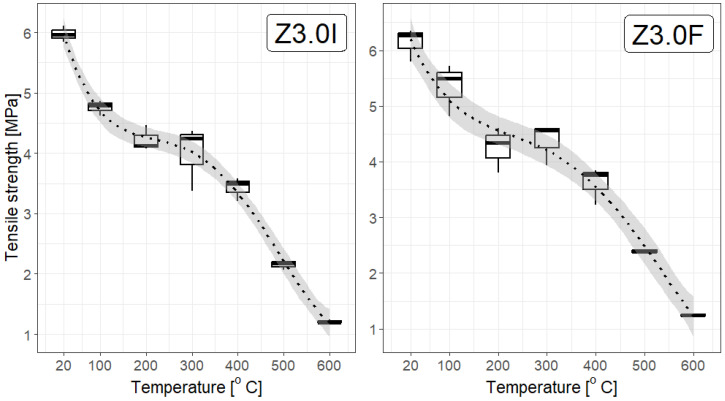
Box plots for the tensile strength of a selected fibre type with a density of 3.0 kg/m^3^. The linear model of decline with temperature poorly reflects the actual relationship (R^2^ below 0.8); therefore, a third-degree polynomial model was used. The coefficient of determination in this case is 0.91, and the 95% confidence interval is narrow.

**Figure 16:**
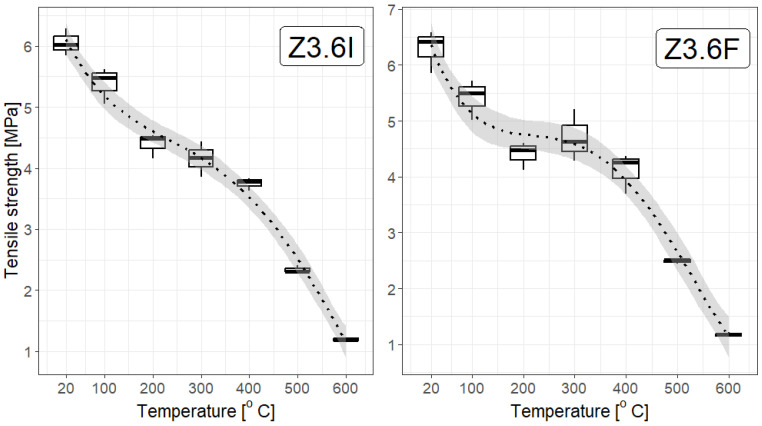
Box plots for the tensile strength of a selected fibre type with a density of 3.6 kg/m^3^. The linear model of decline with temperature poorly reflects the actual relationship (R^2^ approximately 0.8); therefore, a third-degree polynomial model was used. The coefficient of determination in this case is 0.91, and the 95% confidence interval is narrow.

From a statistical point of view, the dependence of the strength of F/I fibre-reinforced mortars on temperature, shown in Figure 14, Figure 15 and Figure 16, constructed for different F/I fibre densities, seems to be interesting. The correlation coefficients are high and suggest a strong, nearly linear dependence, They are as follows: Z0: −0.97, Z1.8F: −0.946, Z1.8I: −0.954, Z3.0F: −0.952, Z3.0I: −0.930, Z3.6F: −0.958, and Z3.6I: −0.981. Figure 14 and Figure 15 clearly show a plateau in the temperature range between approx. 200 °C and 400 °C, which means that, on average, the strength remains constant in this temperature range. This is obviously important during the development of a fire, but mainly in the first three minutes of the first phase. Fitting a linear function to any relationship other than Z0 and Z3.6I gave coefficients of determination below 0.8, which is relatively small.

Instead, a third-degree polynomial model was proposed, which better reflects the observed plateau (due to its saddle point) and provides a good prediction of the strength values at both ends of the studied temperature range. The determination coefficients in all cases reach satisfactory values above 0.9. A typical approach in physical and materials-related problems was used, where linear or polynomial approximation is often employed. If the linear model poorly reflects the behaviour of the system, attempts should be made to approximate it with additional terms from the Taylor series expansion. The first real term in this case is the third-order term, i.e., a function of the form ftm(T) = aT^3^ + bT^2^ + cT + d, where T is the temperature, and a, b, c, and d are the parameters. The narrow 95% confidence interval for all the fitted functions suggests a good choice and statistically significant prognostics regarding the strength values of ftm at different temperatures. A good review article on correlation and regression analysis is provided by [80].

### 3.5. Statistical Analysis of Thermogravimetric Results

A statistical analysis was performed to assess the impact of the amount of PP fibres on the mass loss of mortar heated at a specific temperature. The results of the analysis are presented in Table 14; the tests were performed at significance level α = 0.05.

The results presented in Table 14 show that, in the set of all preheated samples at different temperatures and for both types of fibres (or without additives), on average, the smallest mass loss occurs in samples with fibre addition. This is true both for 1.8 kg/m^3^ and 3.6 kg/m^3^, and in some cases it is even better at the density of 3.6 kg/m^3^. This is particularly evident when comparing the medians, with the best effect observed for mass losses at 100 °C, 200 °C, 300 °C, and 400 °C. It should be noted that each statistical value was calculated for a set of samples with different preheating conditions, ranging from 100 °C to 600 °C. This confirms the conclusions drawn from the thermoanalytical studies, demonstrating that the addition of polypropylene fibres has a positive effect on the thermal stability of the mortars exposed to high temperatures. However, the type and amount of PP fibres in the tested range (1.8 kg/m^3^ vs. 3.6 kg/m^3^) have no statistically significant impact. This applies to thermal stability based on thermogravimetric curves, i.e., mass loss at specific temperatures: W100C, W200C, W300C, W400C, W500C, W600C, and W700C. This effect can be further emphasised by using statistical tests. Since all the variables follow a normal distribution (which was verified using the Shapiro–Wilk test; see *p*-values in Table 13), parametric tests can be applied—in this case, pairwise *t*-tests (for more on parametric tests, see [76]). The null hypothesis for mass loss is that for the density listed on the left (in the column), the mass loss is greater than for the density above (in the row). Rejection in favour of the alternative hypothesis means that, at the given significance level, it can be assumed that this mass loss is smaller. In this study, the FDR adjustment method has been used, which is one of the most balanced measures for the error of I type; as demonstrated in [81] and Table 15.

**Figure 17:**
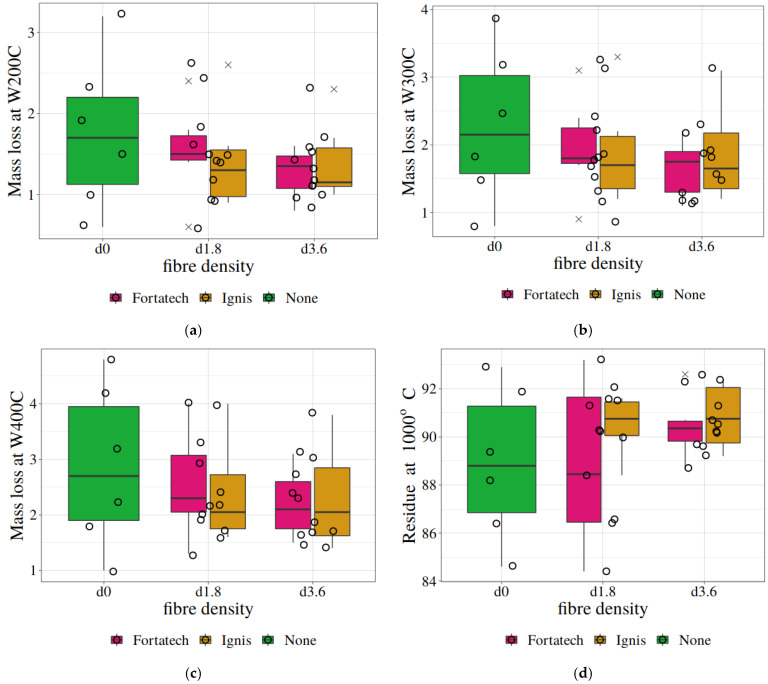
Mass loss at W200C (**a**), W300C (**b**), and W400C (**c**), and residual mass at 1000 °C (**d**) for mortar samples with or without fibre additives at different concentrations. The circles represent individual values for samples preheated at different temperatures. The box plot graphs reflect the basic descriptive statistics of the sample, i.e., the median (horizontal line), and the first and third quartiles, Q1 and Q3 (the lower and upper edges of the rectangle), as well as the minimum and maximum values that are not extreme or outlier values (the lower and upper limits of the “whiskers”). The crosses indicate outlier values (mild outliers, i.e., values which are less than 1.5·(Q_3_ − Q_1_) in the case of lower outliers, or greater than 1.5·(Q_3_ − Q_1_) in the case of upper outliers).

Table 15 shows that at a significance level of 0.20, it can be concluded that for W100C–W300C, the smallest mass loss in the group of samples preheated at different temperatures occurred for mortars with fibres of any type at a density of d3.6. Furthermore, for the density of d1.8, the mass loss was also smaller than for mortars without fibre additives. At higher temperatures, especially from 600 °C onwards, the differences in mass losses no longer seem to be statistically significant. What is more, the residual mass at 1000 °C does not differ statistically, regardless of the fibres used. This reasoning is visually represented in Figure 17, which presents the statistics of the mass loss results at W200C (a), W300C (b), and W400C (c), and the residual mass at 1000 °C (d), in the form of box plots created using R software, ver. 4.5.1 (2025-06-13 ucrt) [73].

At higher temperatures, the statistics are no longer as clear-cut, but it should be borne in mind that they pertain to a set of samples preheated under different temperature conditions. As may be seen below, for samples preheated at a specific temperature, the reasoning is stronger.

**Figure 18:**
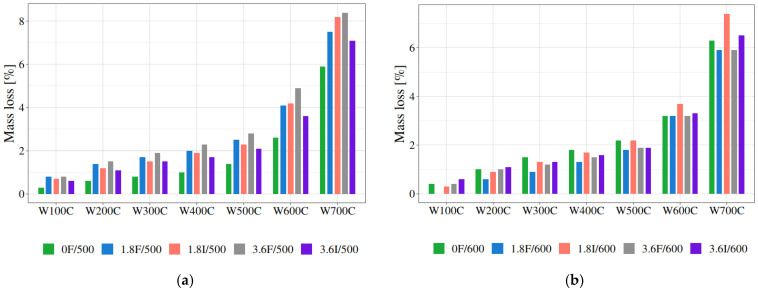
Mass loss for mortar samples preheated at different temperatures: 500 °C (**a**) and 600 °C (**b**). The results are presented for different densities and types of fibres. An inversion of the results compared to lower temperatures is evident.

Figure 10 shows the results for mortar samples heated at a specific temperature. The strongest effect may be seen for samples heated at 200 °C and 300 °C. The mass losses are the lowest for mortars with fibre additives across the entire temperature range in the thermogravimetric analysis. However, starting at 400 °C, the differences between the mass losses for mortars with and without fibre additives disappear, and even worsen for mortars with fibres. This is particularly evident for mortars heated to 500 °C and 600 °C (see Figure 18a,b, which present results for mortars preheated at 500 °C and 600 °C). In this data set, one result was an extreme value (an upper extreme outlier is a value which exceeds three times the interquartile range IQ = Q_3_ − Q_1_, where Q_1_ is the first or lower quartile, and Q_3_ is the third or upper quartile) and it should have been discarded as unreliable: 1.8I/500 had almost a 50% mass loss, which is physically impossible. For more information regarding how to deal with mild and extreme outliers, please refer to [82]. Figure 18b shows the results for mortars preheated at 600 °C, and it is clearly visible that they are no longer as differentiated. In fact, for some types of fibres, the mortars with fibre addition exhibit greater mass losses than those without any additives.

The effects presented in Figure 10 and Figure 18 are further reinforced by statistical data. Pairwise *t*-tests were conducted for the mass loss of samples preheated at a specified temperature, comparing the groups with the densities d0, 1.8F, 3.6F, 1.8I, and 3.6I. The results of this analysis are presented in Table 16.

It can be clearly concluded from Table 16 that the mortar samples with fibre additives, both F and I, preheated at lower temperatures, 100–400 °C, were found to have lower mass losses compared to the mortars without fibres than the samples preheated at higher temperatures, as shown in Figure 18. Although the significance level is not very high, i.e., α = 0.2, it still represents a fairly reliable statistical inference. The fact that a better significance level cannot be obtained stems from the fact that each sample set compared within the group of different densities includes results across a wide temperature range in the thermogravimetric studies: W100C–W700C. As previously noted, at these temperatures, the differences between samples with and without fibres are no longer statistically significant; see Table 16 and Figure 18.

**Figure 19:**
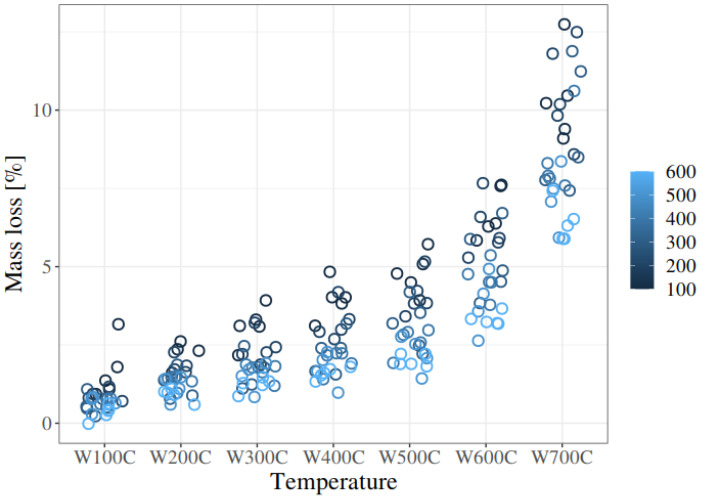
Very well visible variability of the mass loss of every sample studied. This figure presents all the results within each group: W100C, W200C, W300C, W400C, W500C, W600C, and W700C.

Figure 19 contains a summary of all data concerning the mass loss of the mortars with or without fibres, for different pre-firing conditions. Each circle represents one result: the mass loss of the mortar sample. The lighter the circles, the higher the pre-firing temperature of the sample. Two relationships are worth noting: firstly, in thermogravimetric tests, the mass loss increases non-linearly with increasing temperature; secondly, the initial annealing conditions determine the subsequent effects of TG tests, which is namely that the higher the initial annealing temperature, the lower the losses across the entire test range, on average.

## 4. Conclusions

The incorporation of polypropylene fibres proves to be advantageous in terms of thermal analysis and the tensile strength parameter under investigation. The effects of their use are beneficial both at room temperature and in fire conditions.

Statistically significant better tensile strength is achieved with the addition of polymer fibres I/F, with the concentrations of 3.0 and especially 3.6 being the most optimal. Additionally, from a statistical point of view, the mortar with the addition of fibre F shows a slightly better performance, demonstrating superior strength parameters in almost every tested temperature.

The optimal fibre concentration for tensile strength was found to be 3.6 kg/m^3^. The authors conclude that the reason for the better strength of samples with polypropylene I/F fibres, even at high temperatures, is their higher strength at room temperature, which—although decreasing as the temperature rises—remains greater than that of mortars without fibre addition.

Noteworthy is the characteristic plateau in the *f_tm_*(T) function graph, i.e., the tensile strength versus the temperature, especially for the addition of F-type fibres; this shape differs significantly from *f_tm_*(T) for mortar without additives, which decreases linearly in the tested temperature range. This different behaviour suggests a certain inertia in the strength response of mortar with I/F fibre addition as the temperature increases.

The tensile strength results were consistent with thermoanalytical studies where polypropylene fibres in the tested cement composites have a positive effect on the thermal stability of the examined cement mortars. Lower mass losses and higher temperatures of decomposition were observed in samples with PP fibres. Moreover, mortars with fibre addition performed significantly better; yet, statistically, there is no difference between mortars with different types of fibres or varying densities. Therefore, the enhanced thermal stability of the samples with PP fibres in the analysis of spalling mechanisms may also play an important role.

During the fire exposure of fibre-reinforced concrete with PP fibres, the fibres soften, then melt, and partially integrate into the structure of the cement matrix (penetrating into the porous cement matrix), thus ‘reinforcing’ it. PP fibres therefore have an impact in terms of improving tensile strength (in the context of the occurrence of explosive spalling of concrete, a phenomenon in which the tensile strength of the concrete plays a key role), and help to reduce the steam pressure in the pores of the material. As the fibres melt, they penetrate the porous cement matrix, leaving voids that, together with the existing pores in the cement matrix, form an interconnected network. Upon reaching the percolation threshold, this network facilitates the reduction in steam pressure within the material pores. Both of these positive effects of the fibres should be considered together.

The mechanism of action of PP fibres in fibre-reinforced concrete during a fire assumes that at elevated temperatures (above 160 °C), the PP fibres soften and then melt, causing a reduction in the volume of the individual fibres. The voids created by the fibres form channels through which steam escapes under high pressure. As a result, the internal stresses do not reach the critical point, preventing the explosive spalling of the concrete from the structure [9,83,84].

Earlier, our studies showed that the reinforcement of the cement mortar with PP fibres increased its thermal stability after its preliminary heating at 200 °C and 300 °C [55]. This was explained by a combination of dehydration of the hydration products and the melting of the PP fibres after the thermal treatment, which affected the residual permeability of the heated samples and led to changes in the resulting structure. Similar observations were made during this study. This pattern was observed regardless of the kind and amount of polypropylene fibres added after heat treatment at 100, 200, 300, and 400 °C. The positive effect of fibres on mass loss (thermal stability) diminishes or reverses after preheating at 500 °C and 600 °C. It seems that the pre-treatment of mortars at higher temperature influences PP fibre transitions. The explanation for this may be linked to the earlier melting and/or degradation of PP fibres at higher temperatures, which resulted in the lack of PP impact on the thermal stability that was determined by the thermal analysis study.

SEM observations confirmed the absence of damage in the fibre–cement paste contact zone after heating up to 300 °C. The fibres in the tested samples did not undergo complete destruction (pyrolysis). In the mortars heated to 200 °C, 300 °C, and even 400 °C, some fibres underwent surface changes, and fragments of the paste adhered quite tightly to their surfaces (with a visible layer of hydration products around the fibres). Some fibres became covered with a relatively dense layer of paste, creating a kind of fibre–paste contact layer. Therefore, the fibres surrounded by the paste did not undergo pyrolysis, as the temperature at which the thermal decomposition of the tested fibres begins ranges from 385 °C to 415 °C—tested in a nitrogen atmosphere [61].

**Figure 20:**
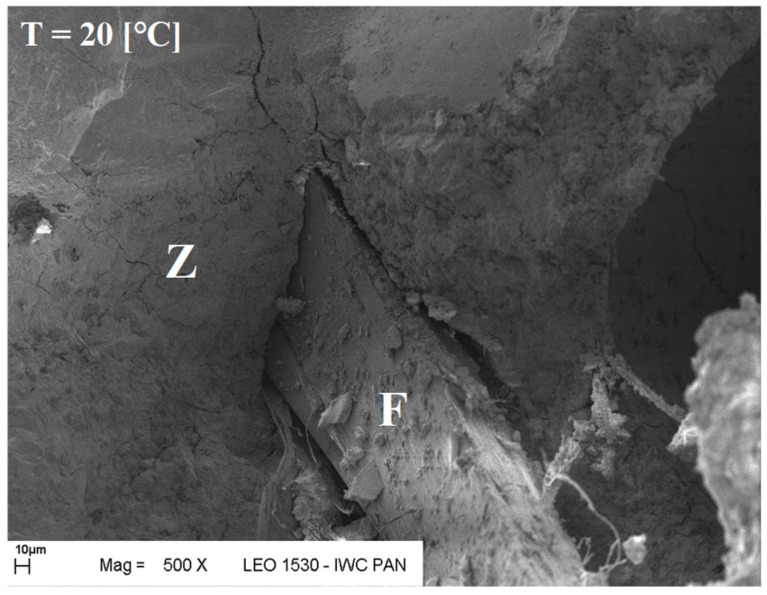
Fibre (F) surrounded by slurry (Z); 20 °C.

**Figure 21:**
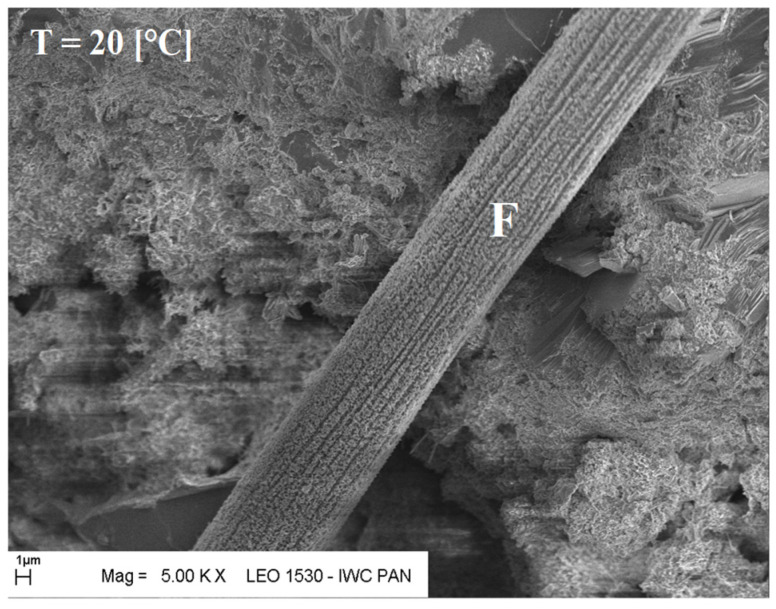
Fibre (F) coated with cement slurry; 20 °C.

**Figure 22:**
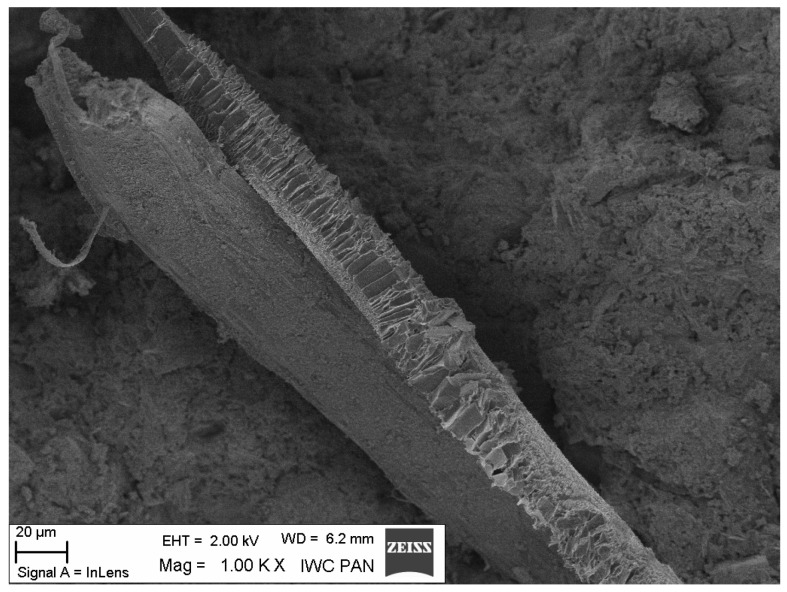
Fibre F in the paste (the fibre covered with a dense layer of paste shows no surface changes, while the fibre without a surface layer of paste shows a clearly altered surface); 200 °C.

**Figure 23:**
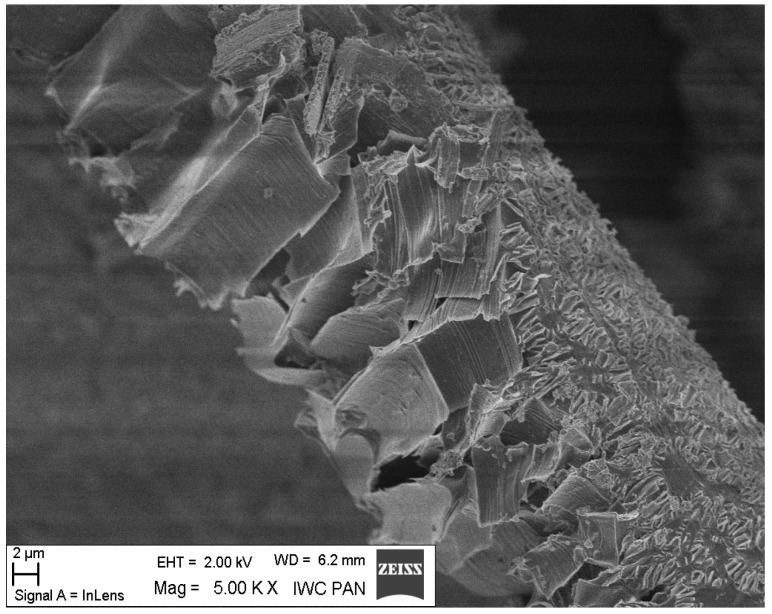
F fibre in the binder (enlarged fragment of fibre surface changes); 200 °C.

**Figure 24:**
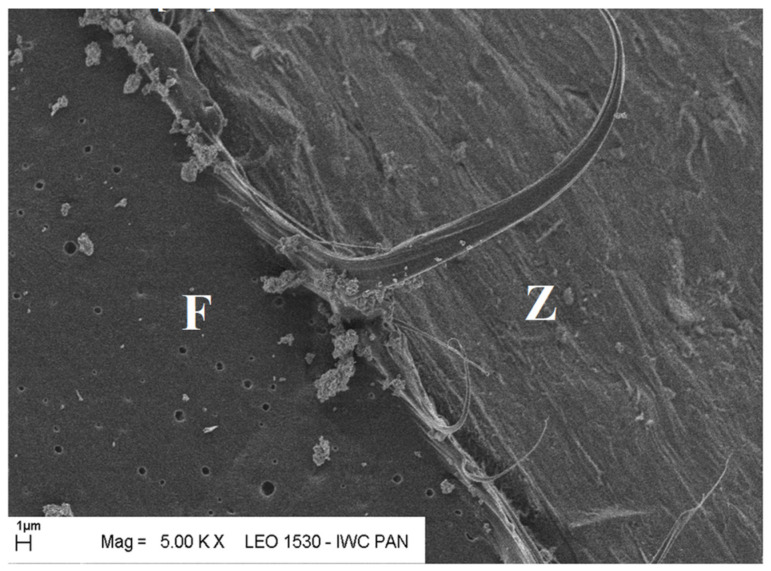
Residue of fibre F and trace of the fibre surrounded by grout (Z); 300 °C.

**Figure 25:**
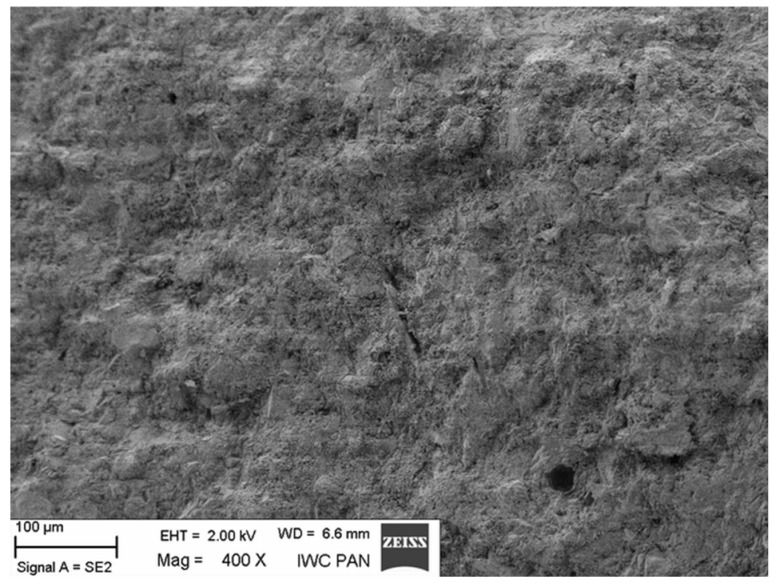
Pores in the structure of the grout; 300 °C.

**Figure 26:**
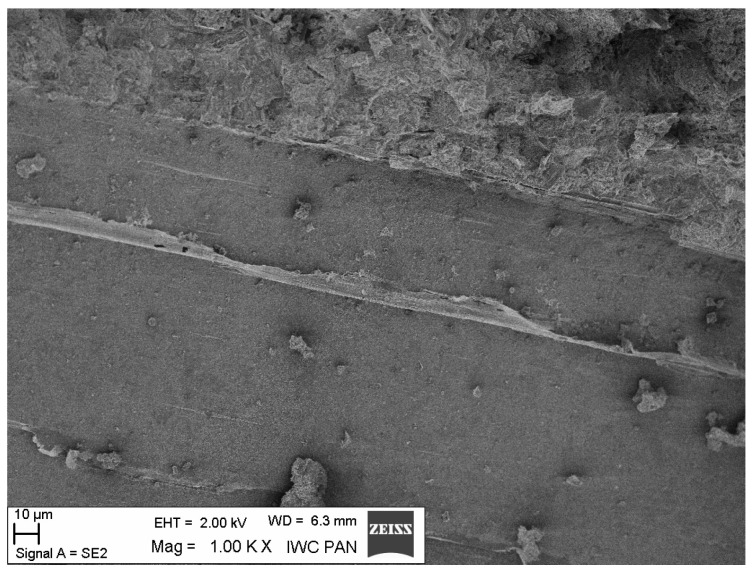
Trace of fibre F in the paste with a visible, fairly dense contact layer; 400 °C.

**Figure 27:**
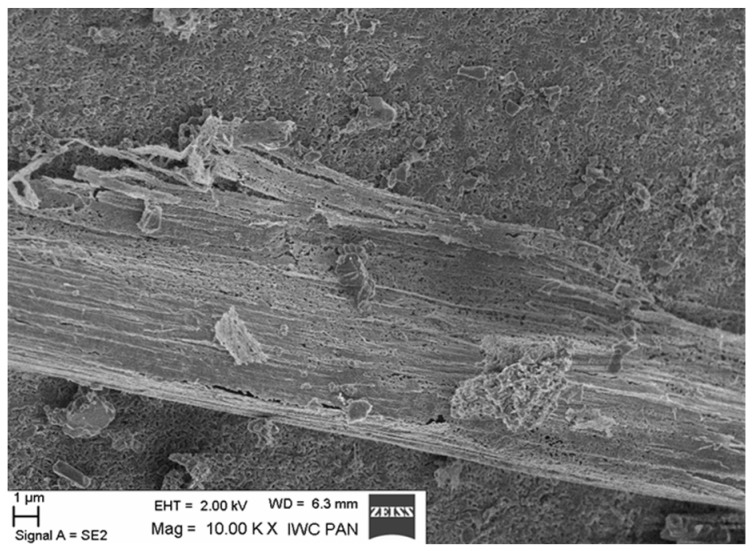
Residue of fibre F in the slurry with visible fine-pored fibre surface; 400 °C.

The microscopic analysis was performed using a scanning electron microscope (SEM), model LEO 1530, manufactured by ZEISS at the Institute of High Pressure Physics of the PAS in Warsaw. The selected SEM images (Figure 20, Figure 21, Figure 22, Figure 23, Figure 24, Figure 25, Figure 26 and Figure 27) show the structure of the cement paste around the polypropylene fibres “F” in the tested samples exposed to high temperatures. Special attention was given to the fibres coated with cement paste.

The SEM analysis has shown that polypropylene fibres undergo gradual degradation within the temperature range of 20 °C to 600 °C. Up to approximately 300 °C, the PP fibres remain intact and serve as dispersed reinforcement in the composite. Their slight loss does not adversely affect the tested tensile and compressive strength [1]. In this temperature range, their influence is beneficial. The dispersed fibres, or even their fragments, positively affect both the transfer of global tensile stresses, which has a beneficial impact on the tensile strength of the composite, and the transfer of local tensile stresses, e.g., around discontinuities, which contribute to the compressive strength of the composite. Above 300 °C, the fibre loss increases significantly until their complete degradation occurs. In terms of tensile strength, even small remnants of fibres can provide beneficial reinforcement, and throughout the entire temperature range, the addition of fibres has a positive impact on this property.

## Figures and Tables

**Table 1:** Physicochemical parameters of CEM I 42.5 R cement based on the technical data sheet.

Property	Unit	Mean Result	Requirements
Beginning of setting	min	233	>60
End of setting	min	291	
Water demand	%	27.5	
Volume stability	mm	1.1	<10
Specific surface area	cm^2^/g	3688	
Compressive strength: after 2 days	MPa	23.9	<10
Compressive strength: after 28 days	MPa	55.9	>42.5 < 62.5
Chemical analysis: SO_3_	%	2.77	<3.0
Chemical analysis: Cl	%	0.070	<0.10
Chemical analysis: Na_2_O eq.	%	0.53	<0.6

**Table 2:** Basic properties of microsilica based on the product data sheet.

Parameter	Unit	Value	Evaluation Method
Form	-	fine-grained powder	visual
Colour	-	Grey	visual
Odour	-	Odourless	-
Density	g/cm^3^	2.05	EN 1097-6 [63]
Bulk density	g/cm^3^	1.1	EN 1097-3 [64]
Alkalinity	pH	lower than 11.5	PN-EN-ISO 10523 [65]

**Table 3:** Basic properties of the admixture based on manufacturer’s data.

Feature	Description
Form	liquid
Colour	light brown
Density	1070 ± 20 kg/m^3^
pH	6.5 ± 1
Contents of Cl^−^	≤0.1%
Contents of Na_2_O	≤1.5%
Raw material base	Polycarboxylic ethers

**Table 4:** Characteristics of Fibrofor High Grade and Ignis fibres used in the tests.

Property	Fibre Name	
	Ignis	Fibrofor High Grade 190
Colour	Transparent	Beige
Characteristic	Monofilament	Bonded, fibrillated
Length, mm	12	19
Film thickness, μm	18	80
Density, g/cm^3^	0.91	0.91
Tensile strength, N/mm^2^	min 28 cN tex^−1 a^	~400
Softening temperature, °C	~165	~150

^a^ cN tex^−1^—tensile strength unit for fibre, where tex is a unit for the linear mass density of fibres.

**Table 5:** Cement mortar compositions.

Components	Abbreviation						
	0F	1.8F	3.0F	3.6F	1.8I	3.0I	3.6I
Cement CEM I 42.5 R, kg/m^3^	846	846	846	846	846	846	846
Silica, kg/m^3^	84.6	84.6	84.6	84.6	84.6	84.6	84.6
Sand, kg/m^3^	1249	1249	1249	1249	1249	1249	1249
Optima 185 plasticiser, % cement mass	2	2	2	2	2	2	2
Water, dm^3^	215	215	215	215	215	215	215
Polypropylene fibres, kg/m^3^	0	1.8	3.0	3.6	1.8	3.0	3.6

**Table 6:** DSC results of polypropylene fibres 207 J/g.

Fibre	I Cycle—Heating		II Cycle—Cooling		III Cycle—Heating	
	Melting Temperature, °C	Melting Enthalpy, J/g	Crystallisation Temperature, °C	Crystallisation Enthalpy, J/g	Melting Temperature, °C	Melting Enthalpy, J/g
I	I peak: 157.9 II peak: 171.1	1.8 55.0	119.5	95.7	161.8	78.8
F	I peak: 126.8 II peak: 171.9	6.3 46.9	115.0	92.6	I peak: 129.6 II peak: 164.0	11.4 57.6

**Table 7:** Thermoanalytical results of polypropylene fibres in air atmosphere.

Fibre	T_5%_	T_10%_	DTG_peak_	DTA_peak1_	DTA_peak2_
Ignis	261.7	276.1	345.2	172.5 (endo)	347.2 (exo)
Fibrofor	272.0	284.6	374.9	169.4 (endo)	375.9 (exo)

**Table 8:** Thermogravimetric results of polypropylene fibre-reinforced mortar samples.

Sample	Mass Loss, W100C, %	Mass Loss, W100–450C, %	Mass Loss, W450–520C, %	Mass Loss, W600–900C, %	Residue, W1000C, %
0F/20	0.98	2.6	0.7	5.1	89.3
1.8F/20	2.7	3.99	0.5	2.97	88.8
3.6F/20	1.5	3.5	0.9	5.9	86.5
1.8I/20	1.4	3.4	0.8	5.5	87.1
3.6I/20	1.3	3.1	0.7	3.9	89.7
0F/100	0.9	3.5	1.0	8.1	84.4
1.8F/100	1.7	3.4	0.8	3.1	89.4
3.6F/100	0.5	2.9	0.8	5.5	88.7
1.8I/100	1.4	3.0	0.6	3.7	89.9
3.6I/100	1.1	3.0	0.7	4.1	89.6
0F/200	0.9	2.7	0.9	7.0	86.4
1.8F/200	1.2	3.5	0.9	6.0	86.4
3.6F/200	0.7	2.3	0.7	5.1	89.7
1.8I/200	0.7	2.4	0.8	5.7	88.4
3.6I/200	0.5	2.3	0.6	4.9	90.2
0F/300	0.8	2.0	1.0	7.5	86.6
1.8F/300	1.0	2.6	1.0	5.1	88.2
3.6F/300	0.2	1.8	0.7	5.3	90.2
1.8I/300	0.5	1.3	0.6	4.6	91.6
0F/400	0.8	1.6	0.8	10.0	84.6
1.8F/400	0.7	1.8	0.6	4.9	90.3
3.6F/400	0.6	1.6	0.6	4.8	90.7
1.8I/400	0.8	1.7	0.6	4.2	91.3
3.6I/400	0.5	1.1	0.6	5.0	91.3
0F/500	0.3	0.9	0.4	5.6	91.9
1.8F/500	0.7	1.4	0.4	3.7	92.1
3.6F/500	0.7	1.7	0.6	4.7	90.5
1.8I/500	0.8	1.5	0.5	5.1	90.6
3.6I/500	0.5	1.3	0.4	4.1	92.3
0F/600	0.4	1.6	0.4	3.9	92.9
1.8F/600	0.0	1.4	0.5	3.6	93.2
3.6F600	0.4	1.2	0.4	4.2	92.6
1.8I/600	0.3	1.6	0.5	4.9	91.5
3.6I/600	0.6	1.3	0.4	4.4	92.1

**Table 9:** Thermogravimetric results of polypropylene fibre-reinforced mortar samples.

Sample	Mass Loss, W100C, %	Mass Loss, W200C, %	Mass Loss, W300C, %	Mass Loss, W400C, %	Mass Loss, W500C, %	Mass Loss, W600C, %	Mass Loss, W700C, %	Residue at 1000 °C, %
0F/100	0.9	2.4	3.1	4.0	5.1	7.6	12.7	84.4
1.8F/100	1.8	3.2	3.9	4.8	5.7	7.6	10.5	89.4
3.6F/100	0.5	1.6	2.2	3.1	3.9	5.8	9.1	88.7
1.8I/100	1.4	2.6	3.3	4.0	4.8	6.3	9.4	90.0
3.6I/100	1.1	2.3	3.1	3.8	4.5	6.4	10.2	89.6
0F/200	0.9	1.8	2.4	3.3	4.2	6.6	11.8	86.4
1.8F/200	0.7	1.4	2.2	2.9	3.6	5.1	8.3	91.3
3.6F/200	0.7	1.4	1.9	2.7	3.4	5.3	8.6	89.7
1.8I/200	0.8	1.6	2.2	2.9	3.8	5.9	10.2	88.4
3.6I/200	0.8	1.7	2.3	3.0	3.8	5.8	9.8	89.2
0F/300	0.8	1.5	1.8	2.4	3.5	5.9	11.9	86.6
1.8F/300	1.1	1.9	2.5	3.2	4.2	6.7	11.2	88.2
3.6F/300	0.2	0.8	1.1	1.7	2.5	4.5	7.8	90.2
1.8I/300	0.5	0.9	1.2	1.6	2.2	3.8	7.4	91.6
3.6I/300	0.5	1.2	1.8	2.4	3.2	4.9	8.5	90.2
0F/400	0.8	1.5	1.8	2.2	3.0	5.4	10.6	84.6
1.8F/400	0.8	1.5	1.8	2.2	2.9	4.8	8.3	90.3
3.6F/400	0.6	1.3	1.6	1.9	2.6	4.5	7.8	90.7
1.8I/400	0.8	1.4	1.9	2.2	2.8	4.5	7.9	91.3
3.6I/400	0.5	1.0	1.2	1.4	1.9	3.8	7.6	91.3
0F/500	0.3	0.6	0.8	1.0	1.4	2.6	5.9	91.9
1.8F/500	0.8	1.4	1.7	2.0	2.5	4.1	7.5	92.1
3.6F/500	0.8	1.5	1.9	2.3	2.8	4.9	8.4	90.5
1.8I/500	0.5	1.0	1.3	1.6	2.0	3.7	7.5	91.3
3.6I/500	0.6	1.1	1.5	1.7	2.1	3.6	7.1	92.3
0F/600	0.4	1.0	1.5	1.8	2.2	3.2	6.3	92.9
1.8F/600	0.0	0.6	0.9	1.3	1.8	3.2	5.9	93.2
3.6F/600	0.4	1.0	1.2	1.5	1.9	3.2	5.9	92.6
1.8I/600	0.3	0.9	1.3	1.7	2.2	3.7	7.4	91.5
3.6I/600	0.6	1.1	1.3	1.6	1.9	3.3	6.5	92.4

**Table 10:** The results of the tensile strength measurement, *f_tm_* [MPa]. The mean values ± standard deviation of the mean for each group of results at the tested temperature and the given fibre density I or F are highlighted in bold.

Temperature, °C	Tensile Strength *f_tm_*, MPa						
	Samples with No Fibres	Samples with Fibres Ignis “I”			Samples with Fibrofor “F” Fibres		
	0.0 kg/m^3^	1.8 kg/m^3^	3.0 kg/m^3^	3.6 kg/m^3^	1.8 kg/m^3^	3.0 kg/m^3^	3.6 kg/m^3^
20 °C	4.44	6.36	5.97	5.84	6.34	6.27	5.85
	5.44	5.94	6.11	6.29	5.45	6.35	6.42
	5.20	5.21	5.85	6.02	5.85	5.80	6.58
mean *f_tm_*, ± std.er., MPa	**5.03 ± 0.30**	**5.84 ± 0.34**	**5.98 ± 0.08**	**6.05 ± 0.13**	**5.88 ± 0.26**	**6.14 ± 0.18**	**6.28 ± 0.23**
100 °C	4.21	3.75	4.86	5.05	5.86	5.49	5.01
	4.38	4.54	4.62	5.62	4.88	4.81	5.50
	4.43	3.87	4.79	5.48	4.21	5.72	5.72
mean *f_tm_*, ± std.er., MPa	**4.34 ± 0.07**	**4.05 ± 0.25**	**4.76 ± 0.07**	**5.38 ± 0.18**	**4.98 ± 0.48**	**5.34 ± 0.28**	**5.41 ± 0.21**
200 °C	3.81	4.40	4.12	4.48	4.31	4.60	4.12
	3.77	4.34	4.46	4.53	4.14	4.34	4.48
	3.88	3.75	4.08	4.16	3.84	3.80	4.60
mean *f_tm_*, ± std.er., MPa	**3.82 ± 0.04**	**4.16 ± 0.22**	**4.22 ± 0.12**	**4.39 ± 0.12**	**4.10 ± 0.14**	**4.25 ± 0.24**	**4.40 ± 0.15**
300 °C	3.86	3.62	4.24	3.86	4.39	3.93	5.20
	3.55	4.02	4.37	4.43	4.42	4.61	4.63
	3.63	4.30	3.37	4.17	3.99	4.57	4.28
mean *f_tm_*, ± std.er., MPa	**3.68 ± 0.10**	**3.98 ± 0.20**	**3.99 ± 0.32**	**4.15 ± 0.17**	**4.27 ± 0.14**	**4.37 ± 0.22**	**4.70 ± 0.27**
400 °C	3.06	3.20	3.58	3.78	3.50	3.23	4.37
	2.92	2.96	3.21	3.83	3.42	3.77	4.26
	3.09	3.30	3.49	3.63	3.30	3.84	3.69
mean *f_tm_*, ± std.er., MPa	**3.02 ± 0.06**	**3.15 ± 0.10**	**3.43 ± 0.12**	**3.75 ± 0.06**	**3.41 ± 0.06**	**3.61 ± 0.20**	**4.11 ± 0.22**
500 °C	2.11	2.09	2.18	2.31	2.10	2.43	2.52
	2.07	2.13	2.22	2.41	2.14	2.35	2.44
	2.05	2.11	2.06	2.29	2.21	2.39	2.51
mean *f_tm_*, ± std.er., MPa	**2.08 ± 0.02**	**2.11 ± 0.02**	**2.15 ± 0.05**	**2.34 ± 0.04**	**2.15 ± 0.04**	**2.39 ± 0.03**	**2.49 ± 0.03**
600 °C	1.14	1.27	1.18	1.19	1.37	1.27	1.22
	1.11	1.15	1.20	1.16	1.30	1.25	1.14
	1.12	1.21	1.24	1.22	1.35	1.22	1.17
mean *f_tm_*, ± std.er., MPa	**1.12 ± 0.01**	**1.21 ± 0.04**	**1.21 ± 0.02**	**1.19 ± 0.02**	**1.34 ± 0.02**	**1.25 ± 0.02**	**1.18 ± 0.03**

**Table 11:** Results of pairwise t-tests with a presumed confidence level of α = 0.05 assuming the alternative hypothesis that the strength is greater for samples with a given fibre additive than for samples without the additive. The values of *p* that indicate the rejection of the null hypothesis in favour of the alternative hypothesis are highlighted in bold. The uncertain results, i.e., when the *p*-value is slightly greater than the assumed confidence level α, are marked in italics. However, at a higher level of significance (e.g., 0.2), the null hypothesis could still be rejected, with a 20% chance of making an error. Therefore, conclusions are rarely drawn at such a level.

Density, kg/m^3^	Temperature, °C	*p*-Value for F	*p*-Value for I
1.8	20	0.79	0.88
	100	~1	0.98
	200	0.69	0.85
	300	*0.18*	~1
	400	*0.15*	0.36
	500	0.82	~1
	600	**0.011**	0.24
3.0	20	0.28	0.25
	100	0.22	0.23
	200	0.73	0.34
	300	0.47	~1
	400	0.33	*0.08*
	500	**0.009**	0.60
	600	**0.028**	0.21
3.6	20	**0.04**	*0.10*
	100	**0.04**	**0.04**
	200	*0.15*	*0.18*
	300	*0.11*	0.32
	400	*0.14*	*0.06*
	500	**0.011**	*0.074*
	600	0.21	*0.17*

**Table 12:** Results of the *t*-test conducted to compare the strength of materials with added fibres of type F or I depending on the concentration, for all tested temperatures combined. Bold font indicates the result when the null hypothesis must be rejected.

Null Hypothesis	*p*-Value for x = F	*p*-Value for x = I
Z1.8x < Z0	**0.003**	0.247
Z3.0x < Z0	**<0.001**	**<0.001**
Z3.6x < Z0	**<0.001**	**<0.001**
Z3.0x < Z1.8x	0.266	0.249
Z3.6x < Z1.8x	**0.027**	**0.006**
Z3.6x < Z3.0x	0.380	**0.018**

**Table 13:** Results of the *t*-test comparing the tensile strength of mortars with fibre F and fibre I at different temperatures. The table shows the *p*-values for each comparison, as well as the results of the F-test for homogeneity of variances (*p* < α is highlighted with bold font, as it results in rejection of the null hypothesis). The values in the third column correspond to the *p*-values from the F-test, indicating whether the variances of the two groups (fibre F and fibre I) are significantly different. For all comparisons, the null hypothesis of equal variances could not be rejected.

Temperature, °C	*p*-Value for F ≤ I	Variance F vs. I
20	0.12	0.70
100	**0.026**	0.78
200	0.54	0.78
300	**0.038**	0.34
400	**0.016**	0.22
500	**0.003**	0.16
600	**0.032**	0.10

**Table 14:** Descriptive statistics and results of the Shapiro–Wilk test for the following variables: mass loss at W100C, W200C, W300C, W400C, W500C, W600C, and W700C, and also for residue at 1000 °C. In all cases, the *p*-value is greater than the accepted significance level α = 0.05; thus, one may assume normality. For more information on tests for normality, please refer to [76].

		Mean	Median	Std. Error	Shapiro–Wilk *p*-Value
W100C	d0	0.86	0.80	0.16	0.917 > α
	d1.8	0.68	0.80	0.11	0.063 > α
	d3.6	0.61	0.60	0.07	0.705 > α
W200C	d0	1.66	1.50	0.24	0.659 > α
	d1.8	1.47	1.50	0.26	0.939 > α
	d3.6	1.33	1.25	0.12	0.283 > α
W300C	d0	2.17	1.90	0.30	0.601 > α
	d1.8	1.90	1.80	0.32	0.914 > α
	d3.6	1.76	1.70	0.17	0.222 > α
W400C	d0	2.74	2.20	0.36	0.324 > α
	d1.8	2.45	2.30	0.44	0.956 > α
	d3.6	2.26	2.10	0.22	0.246 > α
W500C	d0	3.23	3.25	0.55	0.995 > α
	d1.8	3.46	2.90	0.41	0.312 > α
	d3.6	2.88	2.70	0.26	0.276 > α
W600C	d0	5.22	5.65	0.80	0.611 > α
	d1.8	5.30	4.80	0.50	0.313 > α
	d3.6	4.67	4.70	0.30	0.667 > α
W700C	d0	9.9	11.2	1.3	0.080 > α
	d1.8	8.93	8.30	0.60	0.714 > α
	d3.6	8.11	8.10	0.37	0.992 > α
RES.AT 1000 °C	d0	87.8	86.5	1.5	0.116 > α
	d1.8	90.22	90.30	0.61	0.885 > α
	d3.6	90.62	90.35	0.37	0.444 > α

**Table 15:** Comparison between the data groups for the mass loss at a given temperature and the residue at 1000 °C, involving the use of pairwise *t*-tests. This comparison allows us to find whether the mass loss is greater or lesser for samples with or without fibres. For mass loss at W100C, W200C, W300C, W400C, W500C, W600C, and W700C, one would accept only a significance level at 80–85%. When the *p*-value is lesser than α, then the null hypothesis must be rejected in favour of the alternative hypothesis.

Temperature in TG Studies/H_0_	Density	d0	d1.8
W100C H_0_: mass loss within the group in column is greater	d1.8	0.17	--
	d3.6	0.13	0.22
W200C H_0_: mass loss within the group in column is greater	d1.8	0.18	--
	d3.6	0.09	0.25
W300C H_0_: mass loss within the group in column is greater	d1.8	0.19	--
	d3.6	0.10	0.27
W400C H_0_: mass loss within the group in column is greater	d1.8	0.24	--
	d3.6	0.12	0.28
W500C H_0_: mass loss within the group in column is greater	d1.8	0.29	--
	d3.6	0.11	0.22
W600C H_0_: mass loss within the group in column is greater	d1.8	0.37	--
	d3.6	0.15	0.21
W700C H_0_: mass loss within the group in column is greater	d1.8	0.58	--
	d3.6	0.23	0.24
Residue at 1000 °C H_0_: residue within the group in column is less	d1.8	0.73	--
	d3.6	0.39	0.21

**Table 16:** Comparison between the data groups for the mass loss during the TG experiment for samples pre-treated at a given temperature, with the use of pairwise *t*-tests without the adjustment method. This comparison serves to find whether the mass loss is greater or lesser for samples with or without fibres. The accepted significance level was 80–85% (i.e., α equals maximally 0.2). The upper *p*-value refers to the null hypothesis that the mass loss of the sample with added fibres is less than the mass loss of the sample without fibres added. The lower *p*-value, in turn, refers to the null hypothesis that this mass loss is greater. As always, when *p* < α, the null hypothesis must be rejected in favour of the alternative one. The *p*-values for which the null hypothesis are to be rejected have been underlined.

Temperature Pre-Treatment	1.8F vs. d0	3.6F vs. d0	1.8I vs. d0	3.6I vs. d0
100 °C	0.44 0.56	0.17 0.83	0.31 0.69	0.30 0.70
200 °C	0.34 0.66	0.17 0.83	0.24 0.76	0.24 0.76
300 °C	0.20 0.80	0.15 0.85	0.13 0.87	0.24 0.76
400 °C	0.29 0.71	0.32 0.68	0.36 0.64	0.22 0.78
500 °C	0.80 0.20	0.87 0.13	-- --	0.72 0.28
600 °C	0.36 0.64	0.43 0.57	0.56 0.44	0.49 0.51

## Data Availability

The original contributions presented in this study are included in the article. Further inquiries can be directed to the corresponding authors.
